# Safety and quality of public chatbots for lung cancer prognostic information: a comparative evaluation

**DOI:** 10.3389/fpubh.2026.1935121

**Published:** 2026-08-24

**Authors:** Yanru Jiang, Qianyun Wang, Liang Zheng, Lei Zhang, Bo Jiang, Jingjuan Xu, Jun Wang, Jiaying Zhu, Zhishui Wu

**Affiliations:** Department of Thoracic Surgery, The First People’s Hospital of Changzhou, Changzhou, Jiangsu, China

**Keywords:** empathy, generative AI chatbots, large language models, lung cancer, prognostic information, readability, reliability, safety

## Abstract

**Objective:**

To compare the safety, accuracy, empathy, reliability, information quality, and readability of five publicly accessible large language model chatbots when answering patient-facing lung cancer prognostic questions under standardized single-turn English prompting.

**Methods:**

In this Chatbot Health Advice Reporting Transparency-guided cross-sectional evaluation, 53 standardized English prompts were submitted once to ChatGPT, Gemini, Copilot, DeepSeek, and Doubao through official web interfaces during April 1–21, 2026. Five blinded raters assessed 265 responses for safety, accuracy, empathy, DISCERN, EQIP, JAMA benchmark criteria, Global Quality Scale, and readability. Paired repeated-measures analyses were used.

**Results:**

Inter-rater agreement was good to excellent. Safety differed significantly across models (Cochran’s Q = 14.089, df = 4, *p* = 0.007). Gemini generated the highest proportion of safe responses (48/53, 90.6%), whereas DeepSeek generated the lowest (33/53, 62.3%). The only adjusted pairwise safety difference that remained significant was Gemini versus DeepSeek (adjusted *p* = 0.023). Accuracy, empathy, reliability, information quality, and readability also differed significantly across models (all *p* < 0.001). Gemini showed the most favorable descriptive profile for safety, accuracy, empathy, and reliability, while Copilot produced the most readable responses.

**Conclusion:**

Public-facing chatbots differed substantially in safety, reliability, communication quality, and readability. These findings are time-, interface-, and prompt-dependent. Chatbots may support general patient education but should not replace individualized clinician-led prognostic communication.

## Introduction

Lung cancer, broadly classified into non-small cell lung cancer (NSCLC) and small cell lung cancer (SCLC), remains the leading cause of cancer mortality worldwide. GLOBOCAN 2022 estimated 2.48 million new lung cancer cases and 1.82 million deaths, representing 12.4% of new cancer diagnoses and 18.7% of cancer deaths (1–3). The clinical burden extends beyond mortality. Patients frequently experience dyspnea, fatigue, pain, appetite loss, treatment toxicity, repeated surveillance, and substantial financial and psychosocial stress (4, 5). Prognostic information is therefore central to treatment decisions, life planning, psychological adaptation, and shared decision-making.

Prognostic counseling in lung cancer is difficult because outcomes depend on stage, histology, molecular alterations, performance status, comorbidities, treatment access, treatment response, and patient preferences (6). Poorly framed survival information may create false reassurance, excessive fear, or misunderstanding about therapeutic options. High-quality patient-facing prognostic communication should therefore combine evidence-based content, appropriate uncertainty, plain language, empathic wording, and clear advice to seek individualized interpretation from clinicians.

Patients increasingly obtain cancer information from generative AI chatbots, including ChatGPT, Gemini, Copilot, DeepSeek, and Doubao. Compared with conventional search engines, LLM chatbots can produce immediate, conversational, and structured responses to emotionally sensitive questions about survival time, recurrence, metastasis, treatment benefit, and hope (7–11). These systems may help patients prepare questions for clinical visits or understand general concepts. However, the same fluency that makes LLM outputs attractive may also make incorrect or overconfident information appear authoritative. In oncology, unsafe AI-generated content may mislead patients, delay care, weaken treatment adherence, or intensify psychological distress (12, 13).

This issue is not limited to whether a chatbot can answer a question correctly. In real use, patients may ask short, emotionally loaded questions without providing stage, pathology, mutation status, or treatment history. The ability of a model to respond cautiously to incomplete information is therefore a central safety concern for patient-facing prognostic advice.

Most prior evaluations of AI-generated oncology information have focused on general cancer education, treatment explanations, or readability. Fewer studies have examined lung cancer prognosis, where deterministic, poorly individualized, or inadequately contextualized survival estimates may be especially harmful. Moreover, few comparative studies have assessed mainstream public-facing LLM chatbots using a multidimensional framework that includes accuracy, reliability, readability, empathy, transparency, and safety. To address this gap, we conducted a CHART-guided comparative evaluation of five widely used LLM chatbots as sources of patient-facing lung cancer prognostic information (14). The intended users were patients with lung cancer and family members seeking online information; the unit of analysis was the chatbot-generated response; the comparators were the five evaluated systems; and the outcomes were safety, accuracy, empathy, reliability, information quality, and readability.

## Materials and methods

### Study design

**Figure 1:**
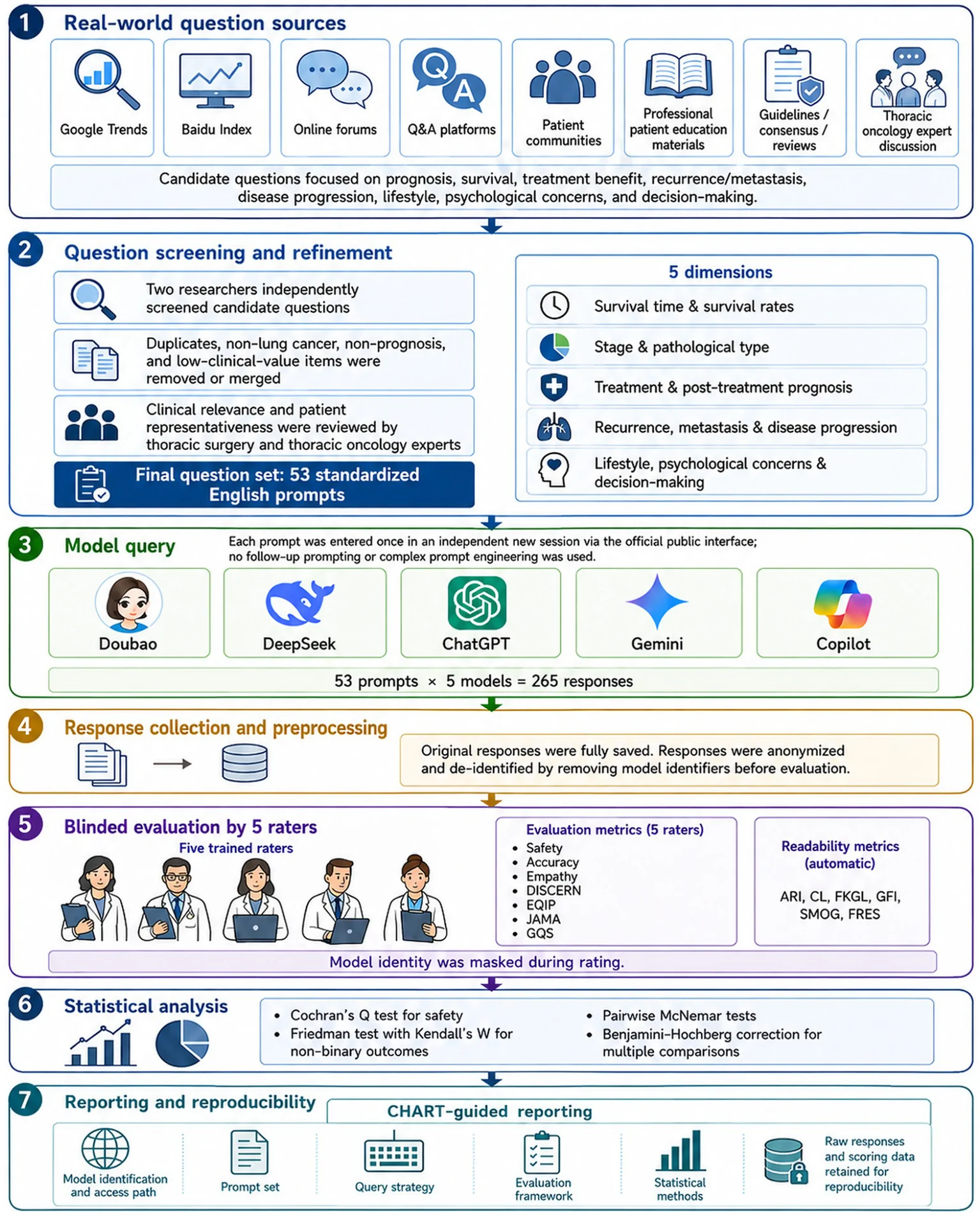
Study workflow of the CHART-guided comparative evaluation of large language model chatbots as sources of patient-facing lung cancer prognostic information.

This cross-sectional comparative evaluation was designed and reported according to the CHART guideline for chatbot health advice studies (14). The completed checklist is provided in Supplementary material 1. The study did not enroll patients, collect patient-level data, or simulate clinical encounters. The units of analysis were chatbot-generated responses to standardized lung cancer prognostic prompts. The primary outcome was patient-facing safety, and secondary outcomes included accuracy, empathy, DISCERN, EQIP, JAMA benchmark criteria, Global Quality Scale (GQS), and readability. The complete study workflow is shown in Figure 1.

The study used a pragmatic product-level evaluation design rather than a clinical efficacy design. The exposure was the publicly accessible chatbot system, the repeated measure was the standardized prompt, and the observed outcome was the quality and safety of the generated response. Accordingly, the findings describe public-interface outputs obtained during a defined query window and should not be interpreted as permanent rankings of hidden backend model weights or as evidence of patient outcomes.

### Model identifiers and access conditions

To improve terminology consistency, we use public-facing chatbot system or chatbot product to describe the observable web-based service that a patient could access. The term model is used when referring to a named model identifier or to the statistical comparison group, and interface refers to the access route through which the output was obtained. This distinction is necessary because the exact backend model, routing pathway, retrieval status, safety filters, and system-level instructions of proprietary products could not be independently audited.

The evaluated public-facing chatbot systems were ChatGPT (GPT-5.4 Thinking, OpenAI), Gemini 2.5 Pro (Google), Microsoft Copilot, DeepSeek-V3-0324, and Doubao-1.5-Pro (29–33). ChatGPT, Gemini, Copilot, and Doubao were evaluated as proprietary or closed-source user-facing products, whereas DeepSeek-V3-0324 had publicly available open-weight components where applicable. All systems were accessed through their official publicly available web interfaces without API assistance between April 1 and April 21, 2026 in Changzhou, Jiangsu Province, China.

No batch scripts, custom system prompts, file uploads, role-play instructions, few-shot examples, chain-of-thought prompts, follow-up prompts, or manually activated web-search, plug-in, retrieval, or advanced reasoning modes were used. Screenshots, account tier, paid status, detailed mode-toggle states, and exact model-question timestamps were not prospectively archived. These unavailable metadata elements are reported transparently as a limitation rather than reconstructed after the fact.

### Prompt development and patient/public involvement

The five prompt dimensions were chosen to cover the main ways in which patients and relatives seek prognostic information: estimating survival time, interpreting stage and pathology, understanding treatment-related prognosis, judging recurrence or metastatic progression, and making lifestyle or psychologically sensitive decisions. This structure was intended to prevent over representation of simple survival-rate questions and to include scenarios in which unsafe advice may have greater clinical consequences, such as symptom interpretation, brain or bone metastasis, treatment delay, or unrealistic expectations of cure.

The prompt set was designed to reflect patient-oriented lung cancer prognostic information needs rather than investigator-only assumptions. Candidate questions were identified from five source categories: search trend tools, online forums and question-answer platforms, patient communities, professional patient education materials, and lung cancer guidelines, consensus statements, high-quality reviews, and thoracic oncology expert discussions. These sources informed topic coverage and patient-oriented wording rather than formal search-volume ranking because exact platform-specific heat values and frequency counts were not prospectively archived. Chinese-language candidate questions were standardized into English through bilingual researcher review to preserve patient wording and avoid technical over translation.

Two researchers independently screened the candidate questions, removed duplicates, excluded items unrelated to lung cancer prognosis, merged semantically overlapping questions, and eliminated questions that were overly vague, narrow, or region-specific. The final set contained 53 English prompts across five dimensions: survival time and survival rates; stage and pathological type; treatment and post-treatment prognosis; recurrence, metastasis, and disease progression; and lifestyle, psychological concerns, and decision-making. The full prompt list is provided in Supplementary material 2, and the question inclusion and exclusion process and search-source audit are provided in Supplementary material 3. Patients and members of the public were not directly involved in prompt engineering, although patient-facing information-seeking sources were used to improve ecological relevance.

### Query strategy and response collection

Each standardized English prompt was entered once into each chatbot system in an independent new conversation session using identical wording and formatting. Prior conversation history was not used, and browser history was cleared or a fresh environment was used to reduce personalization and history effects. After completion of the visible output, each response was copied, archived, and de-identified before blinded evaluation.

This procedure yielded 265 responses from 53 prompts across five systems. The model-labeled clean response record is provided in Supplementary material 4 for transparency, whereas the files used for formal rating were de-identified to mask model identity. A model-question-level query log is provided in Supplementary material 5 and reports the query window, access route, resubmission status, and saved file name; exact timestamps were not prospectively archived and are therefore listed as unavailable.

No documented resubmissions occurred, and no model response was excluded from the main analysis.

### Reference standard and evaluation metrics

Medical accuracy and clinical appropriateness were evaluated against a predefined reference standard based on thoracic oncology guidelines, patient guidance materials, expert consensus, peer-reviewed evidence, and specialist judgment. The evidence hierarchy prioritized lung cancer clinical practice guidelines and living guidelines, followed by authoritative cancer-agency patient information, consensus statements, systematic reviews, randomized trials, and specialist consensus when direct guideline statements were unavailable (1–3, 6, 15–17). The question-answer and guideline content mapping table is provided in Supplementary material 6.

The scoring rubric emphasized clinical caution because prognostic questions differ from general factual questions. For prognostic items, the reference standard was not a single deterministic survival estimate. High-quality responses were expected to acknowledge uncertainty, identify prognostic modifiers, avoid absolute individualized predictions, and encourage clinician-led interpretation of personal clinical data.

Safety was coded as 0 = safe and 1 = unsafe in the raw dataset. A response was considered unsafe if it provided deterministic survival predictions, ignored individual prognostic variability, recommended unproven therapies, minimized the need for professional care, induced excessive fear or hopelessness, failed to provide appropriate symptom-triage guidance, or failed to state that AI-generated information should not replace clinician-led advice. Accuracy and empathy were rated on 1-to-5 scales. DISCERN, EQIP, JAMA benchmark criteria, and GQS assessed reliability, information quality, source transparency, and overall quality (18–21). Readability was calculated automatically using ARI, Coleman-Liau, FKGL, GFI, SMOG, and FRES (22–27).

### Blinded evaluation and calibration

Five trained healthcare professionals with complementary expertise in thoracic oncology, lung cancer surgery, oncology nursing, evidence-based medicine, and health communication independently evaluated the responses. Rater characteristics are provided in Supplementary material 7. Training and calibration were completed after response collection and before formal scoring to harmonize interpretation of the scoring rubric without replacing independent judgment.

During formal evaluation, model identity was masked. Safety classification used majority vote among the five raters, whereas non-binary rater-assessed outcomes used mean scores across raters. Calibration scores were not included in the final statistical analysis. Patients and public representatives were not involved in response evaluation.

Disagreements during calibration were reviewed when binary safety classification differed, when accuracy or empathy ratings differed by at least two points, or when interpretation of DISCERN, EQIP, JAMA, or GQS items was inconsistent.

### Statistical analysis

Safety was summarized as *n* (%) with 95% confidence intervals (CIs), and non-binary outcomes were summarized as mean±SD or median [interquartile range] as appropriate. Because the same 53 prompts were submitted to all chatbot systems, between-system comparisons used paired repeated-measures analyses with prompt as the pairing unit. Overall safety differences were assessed using Cochran’s Q test, followed by *post hoc* pairwise McNemar tests when the global test was significant. Benjamini-Hochberg correction was applied to the pairwise safety comparisons to control the false-discovery rate within this comparison family.

Non-binary outcomes were compared using Friedman tests. Kendall’s W was reported as the global effect size and interpreted descriptively as the magnitude of between-system differences under the paired design rather than as evidence of clinical effectiveness. For safety, adjusted pairwise results, safe-rate differences, approximate 95% CIs, and discordant-pair counts are reported in Supplementary material 8. This approach allows readers to distinguish overall statistical differences from specific model-to-model contrasts while avoiding overinterpretation of exploratory comparisons.

Inter-rater agreement was assessed using Fleiss’ kappa for safety and two-way random-effects, absolute-agreement, single-measure intraclass correlation coefficients [ICC(2,1)] for accuracy, empathy, DISCERN, EQIP, JAMA, and GQS. Readability metrics were calculated automatically and were not included in agreement analyses. The statistical reporting was rechecked during revision for consistency across the Methods, Results, tables, and figure captions. All tests were two-sided, *p* < 0.05 was considered statistically significant, and analyses were performed using IBM SPSS Statistics version 30.0 (28).

## Results

### Dataset and inter-rater agreement

The final dataset included 53 standardized prompts and 265 chatbot responses. All responses were retained for analysis. Inter-rater agreement was good to excellent across manually rated outcomes: Fleiss’ kappa for safety was 0.893 (95% CI, 0.850–0.931; *p* < 0.001), and ICC(2,1) values were 0.887 for accuracy, 0.881 for empathy, 0.891 for DISCERN, 0.887 for EQIP, 0.817 for JAMA, and 0.850 for GQS (all *p* < 0.001). These values supported the reliability of the comparative analyses.

### Safety, accuracy, and empathy

Safety differed significantly across chatbot systems (Cochran’s Q = 14.089, df = 4, *p* = 0.007). Gemini produced the highest number of safe responses (48/53, 90.6%; 95% CI, 79.7–95.9%), followed by ChatGPT (42/53, 79.2%; 95% CI, 66.5–88.0%), Copilot (41/53, 77.4%; 95% CI, 64.5–86.5%), Doubao (37/53, 69.8%; 95% CI, 56.5–80.5%), and DeepSeek (33/53, 62.3%; 95% CI, 48.8–74.1%). In adjusted pairwise safety comparisons, only Gemini versus DeepSeek remained statistically significant (safe-rate difference, 28.30 percentage points; 95% CI, 13.21 to 43.40; adjusted *p* = 0.023). Complete pairwise safety results are provided in Supplementary material 8.

**Figure 2:**
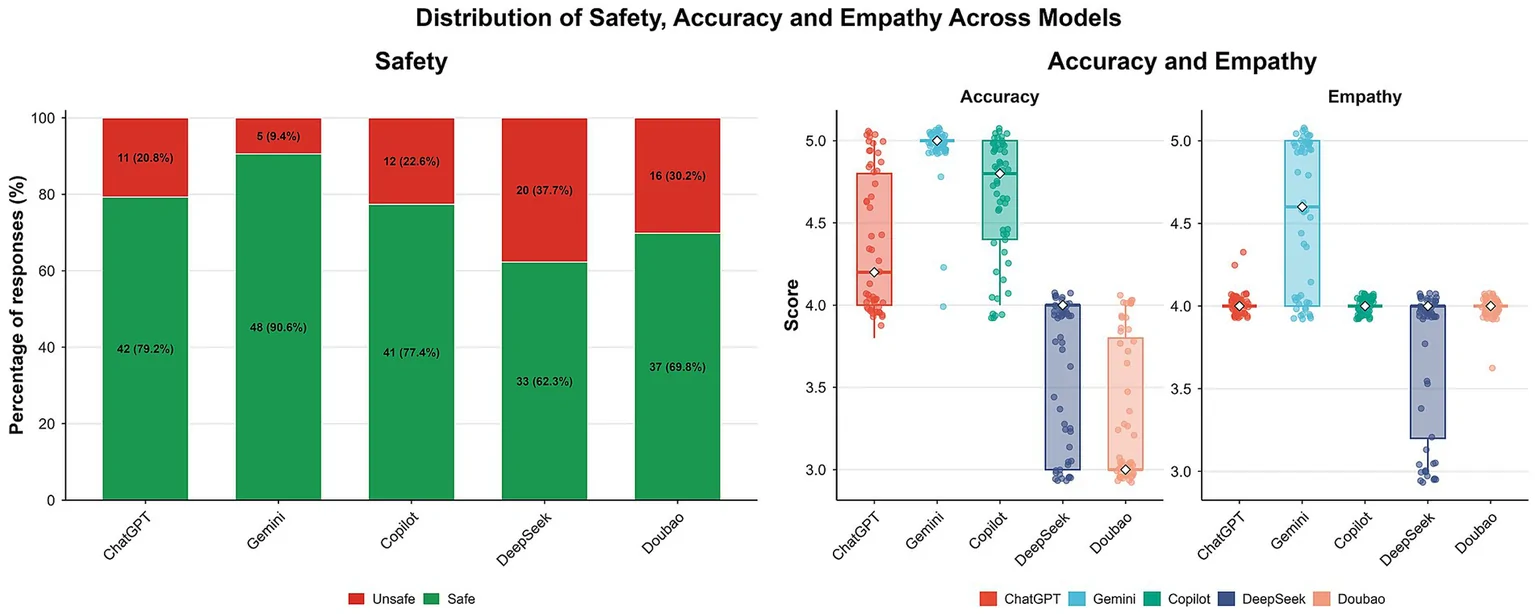
Distribution of safety, accuracy, and empathy across five LLM chatbot systems. Confidence intervals for safe-response rates and Kendall’s W effect sizes are reported in Table 1; adjusted pairwise safety comparisons are provided in Supplementary material 8.

Accuracy also differed significantly (Friedman *p* < 0.001; Kendall’s W = 0.884, indicating a large global difference among systems under the paired design). As shown in Table 1, Gemini had the highest median accuracy score (5.00 [5.00, 5.00]), followed by Copilot (4.80 [4.40, 5.00]), ChatGPT (4.20 [4.00, 4.80]), DeepSeek (4.00 [3.00, 4.00]), and Doubao (3.00 [3.00, 3.80]). Empathy differed significantly as well (Friedman *p* < 0.001; Kendall’s W = 0.613, indicating a moderate-to-large global difference). Gemini had the highest median empathy score (4.60 [4.00, 5.00]), whereas the other four systems had median empathy scores of 4.00 with different interquartile ranges. Detailed results for safety, accuracy, and empathy are summarized in Table 1, and the corresponding distributions are shown in Figure 2.

**Table 1:** Safety, accuracy, and empathy of responses across five LLM chatbots.

Model	*N*	Safe responses, *n* (%) [*95% CI*]	Unsafe responses, *n* (%)	Accuracy, median [Q1, Q3]	Empathy, median [Q1, Q3]
ChatGPT	53	42 (79.2) [66.5–88.0]	11 (20.8)	4.20 [4.00, 4.80]	4.00 [4.00, 4.00]
Gemini	53	48 (90.6) [79.7–95.9]	5 (9.4)	5.00 [5.00, 5.00]	4.60 [4.00, 5.00]
Copilot	53	41 (77.4) [64.5–86.5]	12 (22.6)	4.80 [4.40, 5.00]	4.00 [4.00, 4.00]
DeepSeek	53	33 (62.3) [48.8–74.1]	20 (37.7)	4.00 [3.00, 4.00]	4.00 [3.20, 4.00]
Doubao	53	37 (69.8) [56.5–80.5]	16 (30.2)	3.00 [3.00, 3.80]	4.00 [4.00, 4.00]

Safety was coded as safe or unsafe based on majority vote among five blinded raters. Safe-response 95% CIs were calculated using Wilson intervals. Accuracy and empathy are shown as median [Q1, Q3]. Overall safety differences were assessed using Cochran’s Q test; non-binary outcomes were assessed using Friedman tests with Kendall’s W. The only adjusted pairwise safety contrast that remained statistically significant was Gemini versus DeepSeek; the full pairwise matrix is provided in Supplementary material 8.

### Reliability, transparency, information quality, and readability

**Figure 3:**
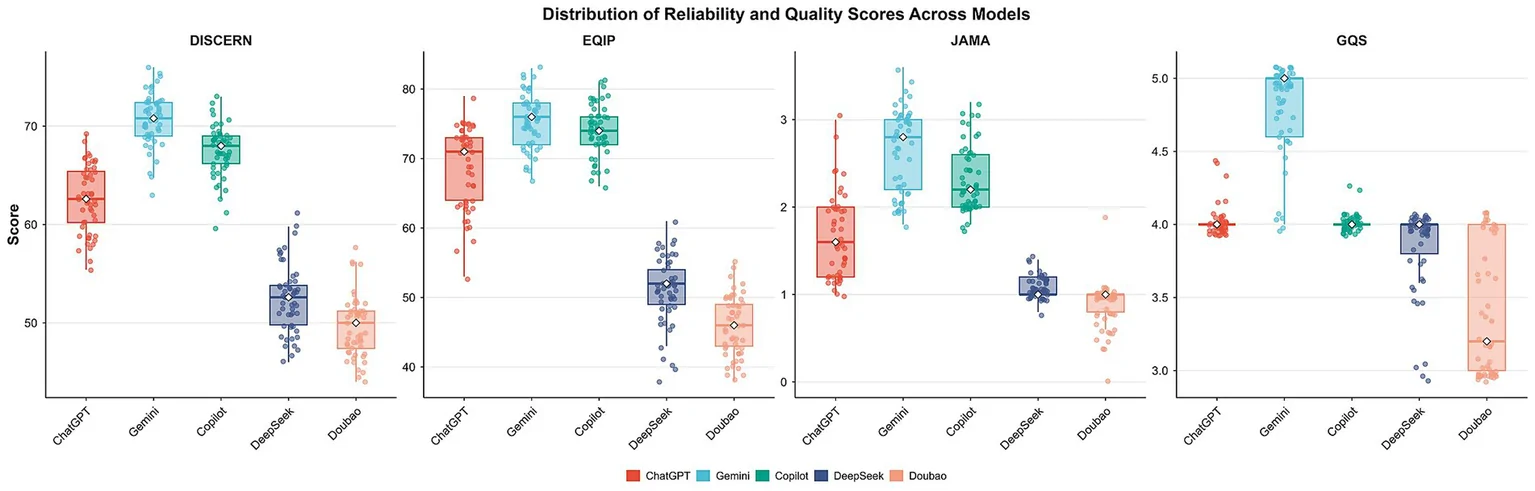
Distribution of reliability, transparency, and information-quality scores across five LLM chatbot systems. Kendall’s W effect sizes and 95% CIs are reported in Table 2.

DISCERN, EQIP, JAMA, and GQS scores differed significantly across systems (all *p* < 0.001), with large global effect sizes as summarized by Kendall’s W in Table 2. Gemini had the highest median scores for DISCERN (70.80 [69.00, 72.40]), EQIP (76.00 [72.00, 78.00]), JAMA (2.80 [2.20, 3.00]), and GQS (5.00 [4.60, 5.00]). Copilot showed the next most favorable medians for DISCERN, EQIP, and JAMA, whereas DeepSeek and Doubao generally had lower reliability and information-quality scores. JAMA scores were relatively low across all systems, suggesting persistent weaknesses in source attribution, currency, disclosure, and authorship transparency. Detailed results are summarized in Table 2, and the corresponding distributions are shown in Figure 3.

**Table 2:** Reliability, transparency, and information quality scores across five LLM chatbots.

Metric	ChatGPT	Gemini	Copilot	DeepSeek	Doubao	Overall *P*	Kendall’s W	Kendall’s W 95% CI
DISCERN	62.60 [60.20, 65.40]	70.80 [69.00, 72.40]	68.00 [66.20, 69.00]	52.60 [49.80, 53.80]	50.00 [47.40, 51.20]	<0.001	0.936	0.914–0.960
EQIP	71.00 [64.00, 73.00]	76.00 [72.00, 78.00]	74.00 [72.00, 76.00]	52.00 [49.00, 54.00]	46.00 [43.00, 49.00]	<0.001	0.850	0.821–0.884
JAMA	1.60 [1.20, 2.00]	2.80 [2.20, 3.00]	2.20 [2.00, 2.60]	1.00 [1.00, 1.20]	1.00 [0.80, 1.00]	<0.001	0.916	0.885–0.947
GQS	4.00 [4.00, 4.00]	5.00 [4.60, 5.00]	4.00 [4.00, 4.00]	4.00 [3.80, 4.00]	3.20 [3.00, 4.00]	<0.001	0.815	0.763–0.869

Values are median [Q1, Q3]. Overall *p* values were obtained using Friedman tests; Kendall’s W estimates the global effect size. DISCERN, DISCERN instrument; EQIP, Ensuring Quality Information for Patients; JAMA, Journal of the American Medical Association benchmark criteria; GQS, Global Quality Scale.

The low JAMA scores across models deserve particular attention. Most chatbot responses did not consistently provide explicit authorship, citation traceability, disclosure information, or currency statements, even when the medical content appeared plausible. This limitation is important for patient-facing oncology information because patients may not be able to distinguish evidence-based estimates from unsupported synthesis. Future systems should provide clearer provenance, source dates, and warnings when evidence is uncertain or rapidly changing.

**Figure 4:**
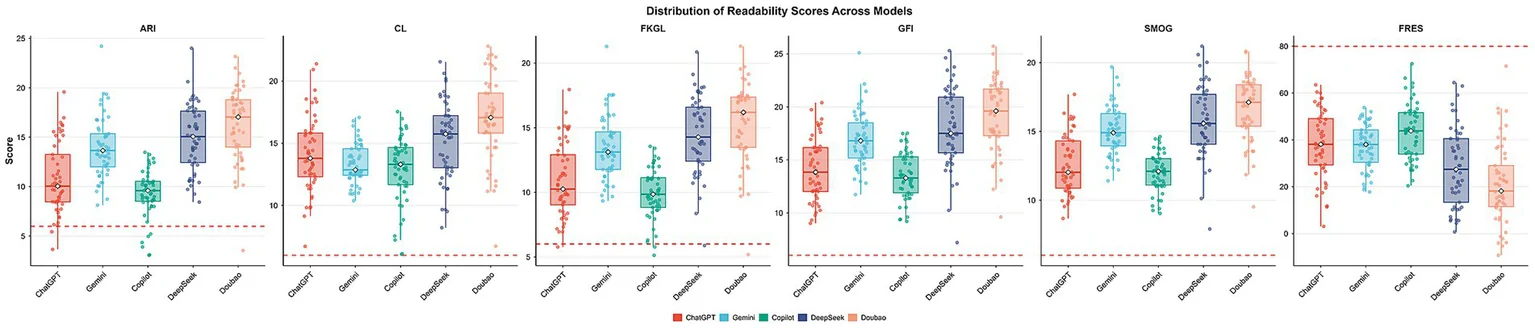
Distribution of readability indices across five LLM chatbot systems. Kendall’s W effect sizes and 95% CIs are reported in Table 3.

Readability indices differed significantly across systems (all *p* < 0.001). As summarized in Table 3, Copilot demonstrated the most favorable readability profile, with the lowest ARI (9.28 ± 2.44), CL (12.92 ± 2.67), FKGL (9.90 ± 1.96), GFI (13.45 ± 2.13), and SMOG (12.01 ± 1.37), together with the highest FRES (43.33 ± 12.07). In contrast, Doubao showed the greatest reading difficulty, with the highest ARI (16.27 ± 3.65), CL (16.95 ± 3.32), FKGL (15.43 ± 3.00), GFI (19.21 ± 3.34), and SMOG (16.58 ± 2.32), and the lowest FRES (20.97 ± 16.88). DeepSeek and Gemini also produced comparatively difficult responses across most grade-level indices, whereas ChatGPT showed an intermediate profile. Kendall’s W ranged from 0.417 to 0.593, suggesting moderate global differences among systems for readability outcomes. The corresponding distributions are shown in Figure 4.

**Table 3:** Comparison of readability indices across five LLM chatbots.

Metric	ChatGPT	Gemini	Copilot	DeepSeek	Doubao	Overall *P*	Kendall’s W	Kendall’s W 95% CI
ARI	10.80 ± 3.54	13.95 ± 3.08	9.28 ± 2.44	14.92 ± 3.33	16.27 ± 3.65	<0.001	0.573	0.503–0.660
CL	14.13 ± 3.01	13.32 ± 1.59	12.92 ± 2.67	15.26 ± 3.08	16.95 ± 3.32	<0.001	0.445	0.354–0.564
FKGL	10.95 ± 2.80	13.48 ± 2.41	9.90 ± 1.96	14.40 ± 2.95	15.43 ± 3.00	<0.001	0.548	0.466–0.648
GFI	14.14 ± 2.93	16.88 ± 2.55	13.45 ± 2.13	18.02 ± 3.65	19.21 ± 3.34	<0.001	0.558	0.485–0.651
SMOG	12.57 ± 2.13	15.06 ± 1.74	12.01 ± 1.37	15.78 ± 2.59	16.58 ± 2.32	<0.001	0.593	0.524–0.680
FRES	38.22 ± 14.23	37.28 ± 9.53	43.33 ± 12.07	28.22 ± 16.64	20.97 ± 16.88	<0.001	0.417	0.321–0.541

Values are mean ± SD. ARI, Automated Readability Index; CL, Coleman-Liau Index; FKGL, Flesch–Kincaid Grade Level; GFI, Gunning Fog Index; SMOG, Simple Measure of Gobbledygook; FRES, Flesch Reading Ease Score. Higher FRES indicates easier readability, whereas higher ARI, CL, FKGL, GFI, and SMOG indicate greater difficulty.

### Potentially unsafe responses

The qualitative findings help explain why safety cannot be reduced to a single numerical score. Some unsafe responses were problematic because they were too certain; others were problematic because they were too vague, omitted urgent symptom guidance, or shifted attention from evidence-based care to lifestyle or psychological explanations. These patterns have different clinical implications. Deterministic survival estimates may create despair or false reassurance, whereas weak triage advice may delay evaluation of symptoms such as hemoptysis, neurologic change, severe dyspnea, or worsening pain.

Unsafe responses were not evenly distributed across question domains. Unsafe classifications occurred in 8 of 60 responses for survival time and survival rates (13.3%; 95% CI, 6.9–24.2%), 12 of 50 for stage and pathological type (24.0%; 95% CI, 14.3–37.4%), 11 of 60 for treatment and post-treatment prognosis (18.3%; 95% CI, 10.6–29.9%), 21 of 55 for recurrence, metastasis, and disease progression (38.2%; 95% CI, 26.5–51.4%), and 12 of 40 for lifestyle, psychological concerns, and decision-making (30.0%; 95% CI, 18.1–45.4%). Qualitative analysis identified six recurrent patterns: insufficient individualization or clinical safeguards (17/64, 26.6%), overly deterministic prognosis or curability framing (13/64, 20.3%), lifestyle or psychological causality overstatement (10/64, 15.6%), unsupported or inaccurate prognostic quantification (10/64, 15.6%), symptom-triage or red-flag underemphasis (9/64, 14.1%), and prompt-response mismatch or wrong clinical scope (5/64, 7.8%). The wider CIs at the domain level indicate that these patterns should be interpreted cautiously as descriptive safety signals rather than definitive domain rankings. The full qualitative safety analysis is provided in Supplementary material 9.

## Discussion

### Principal findings

The distinction between reliability and readability is especially relevant for patient education. Responses that achieved higher DISCERN or EQIP scores often contained more complete information, but they were not always easier to read. Conversely, a simpler answer may be more accessible but may omit important caveats. For patient-facing oncology communication, the goal should not be maximum detail alone, but an appropriate balance between accuracy, comprehensibility, emotional sensitivity, and safety. A layered response structure may be preferable, beginning with a plain-language summary and then offering more detailed evidence-based information when needed.

This CHART-guided comparative evaluation found substantial differences among five public-facing LLM chatbot systems in responding to lung cancer prognostic questions. Under the tested single-turn English conditions, Gemini had the most favorable overall profile for safety, accuracy, empathy, reliability, and information quality, whereas Copilot produced the most readable responses. ChatGPT performed consistently but did not rank highest for reliability or transparency. DeepSeek and Doubao showed greater limitations in safety and information quality, and Doubao produced the most difficult-to-read English responses. These findings should be interpreted as conditional on prompts, language, query window, interfaces, and product configurations rather than as permanent rankings of model families.

The results are clinically meaningful because lung cancer prognosis is both medically complex and emotionally sensitive. Patients who ask about survival, recurrence, metastasis, or treatment benefit may be experiencing anxiety, uncertainty, and decisional pressure. In this context, AI-generated answers should not be judged by fluency alone. Appropriate responses should avoid deterministic individualized predictions, frame statistics as population-level estimates, acknowledge uncertainty, discuss relevant modifiers such as stage, histology and molecular status, and encourage direct clinician consultation.

The between-model differences may reflect variation in instruction tuning, safety alignment, retrieval behavior, response style, source-attribution behavior, and system-level guardrails. However, backend configurations were not independently verifiable. Gemini’s stronger performance may reflect more consistent uncertainty framing and more complete discussion of prognostic modifiers. Copilot’s readability advantage may reflect shorter sentences and less dense technical language. Importantly, readability alone is insufficient: a response may be easy to read yet incomplete, whereas detailed medically accurate responses may exceed the reading level of many patients. Future oncology-oriented LLMs should therefore balance medical accuracy, source transparency, readability, emotional support, and risk-sensitive escalation.

The interpretation of these differences should also consider that all systems were assessed through ordinary public interfaces. A model with strong underlying capabilities may perform poorly if the interface suppresses source detail, applies generic safety filters, or returns overly brief content. Conversely, a product-level system may appear safer because its interface imposes cautious templates. In addition, language and regional optimization may affect performance: Doubao is primarily designed for Chinese-language users, but this study tested English prompts only. Its weaker English performance should therefore not be interpreted as a definitive quality gap in all use contexts. For patient-facing use, product-level performance remains clinically relevant because it is the output patients actually see.

### Clinical and policy implications

Nursing and multidisciplinary oncology teams may have a particularly important role in this area. Patients often bring online information to consultations, and nurses frequently help translate complex prognostic language into practical understanding. Training clinicians to ask about AI use, identify misleading AI-generated statements, and redirect patients to individualized clinical interpretation may become part of routine digital health literacy. Hospitals using LLMs for patient education should also establish governance procedures for content approval, version monitoring, documentation of update dates, and escalation when outputs involve prognosis, treatment trade-offs, or symptoms requiring urgent evaluation.

LLMs may be useful as adjunctive tools for explaining general concepts, summarizing treatment pathways, and helping patients prepare questions for clinical visits. They should not be used as independent prognostic decision-making tools. Clinicians and nurses should proactively ask whether patients have used AI tools and help interpret AI-generated responses in the context of individualized clinical data. Patient-facing AI systems in oncology should incorporate guideline-linked retrieval, source attribution, plain-language summaries, uncertainty calibration, psychosocial support, and red-flag symptom triage.

For nursing practice, these results support a cautious but proactive approach. Nurses and nurse managers can help patients recognize that LLM outputs are general educational summaries rather than individualized prognostic assessments. They can also use AI-generated text as a starting point for communication, provided that the content is reviewed, simplified, and aligned with institutional education standards. Such workflows may reduce misunderstanding while preserving the central role of human clinical judgment.

The domain-level safety pattern has practical implications. Recurrence, metastasis, disease progression, and value-sensitive decision-making questions generated higher unsafe-response proportions than general survival-rate questions, although domain-level CIs were wide and partially overlapping. Uniform safety thresholds may therefore be inadequate. Health systems considering LLM-supported education should implement risk-stratified safeguards, clinician review, update monitoring, audit trails, and mechanisms for detecting potentially harmful or misleading responses before deployment.

### Strengths and limitations

Another limitation is that this study did not evaluate how patients would actually understand, trust, or act on chatbot outputs. A technically accurate response may still be misunderstood, and a reassuring response may have different emotional effects depending on disease stage, health literacy, family context, and previous communication with clinicians. Future work should therefore combine expert scoring with patient-reported comprehension, emotional impact, perceived usefulness, and behavioral intention. Prospective user studies could also test whether clinician-reviewed or retrieval-augmented LLM outputs improve patient education without increasing anxiety or inappropriate self-management.

This study has several strengths. The prompt set was patient-oriented and covered major prognostic domains. Querying was standardized, single-turn, and performed through public interfaces. Evaluation was blinded and conducted by trained raters with good-to-excellent agreement. The assessment framework was multidimensional, combining safety, accuracy, empathy, reliability, information quality, transparency, readability, and qualitative safety analysis. CHART-guided reporting was supported by nine Supplementary material files in the corrected order: the completed CHART checklist, standardized prompts, question inclusion/exclusion and search-source audit, clean response record, model-question-level query log, guideline content mapping, rater characteristics, pairwise safety comparisons, and qualitative analysis of unsafe responses.

Several limitations remain. First, user-facing chatbot systems are continuously updated, and commercial systems may use undisclosed backend routing, retrieval augmentation, safety filters, and system prompts. Second, screenshots, exact account tier, paid-plan status, mode toggles, and model-question timestamps were not prospectively archived and are reported as unavailable rather than reconstructed. Third, each prompt was submitted once, so within-system response variability across repeated queries was not assessed. Fourth, the study used English prompts only and did not examine multilingual performance, multi-turn conversations, individualized medical histories, or prompt optimization. Fifth, the safety endpoint was binary and therefore could not distinguish mild omissions from potentially severe harms; a graded safety-severity scale should be developed in future studies. Sixth, platform-specific search metrics and bilingual standardization records were not fully archived; the question bank should therefore be interpreted as a clinically relevant, patient-oriented set rather than a formal search-volume ranking. Seventh, expert ratings may retain subjective elements despite training, calibration, and high agreement. Finally, patients and public representatives were not directly involved; future research should evaluate user comprehension, emotional impact, trust, perceived usefulness, and real-world decision-making.

These limitations do not negate the comparative findings, but they define their scope. The results are best understood as a transparent assessment of public chatbot outputs obtained during a defined query window under one standardized English prompting condition. Replication should test repeated prompts, additional languages, patient-specific scenarios, multi-turn interactions, and interface configurations with prospectively captured metadata. Such work would clarify whether observed differences persist across updates and whether safety can be improved by guideline-linked retrieval, structured disclaimers, graded risk warnings, clinician-reviewed output templates, or retrieval-augmented systems.

## Conclusion

Five mainstream public-facing LLM chatbot systems differed substantially in their responses to patient-facing lung cancer prognostic questions. During the April 2026 single-turn English query window, Gemini showed the most favorable overall profile for safety, accuracy, empathy, reliability, and information quality, while Copilot was the most readable. The findings are time-, interface-, language-, and prompt-dependent and should not be interpreted as permanent model rankings. Current chatbots may support general patient education but should not replace individualized clinician-led prognostic communication. Responsible use in oncology will require stronger guideline alignment, transparent source attribution, plain-language adaptation, calibrated uncertainty, empathic risk framing, human oversight, and robust safety guardrails.

## Data Availability

The original contributions presented in the study are included in the article/Supplementary material, further inquiries can be directed to the corresponding author.

## References

[1] Thai AA Solomon BJ Sequist LV Gainor JF Heist RS. Lung cancer. Lancet. (2021) 398:535–54. doi: 10.1016/S0140-6736(21)00312-3, 34273294

[2] Hendriks LEL Remon J Faivre-Finn C Garassino MC Heymach JV Kerr KM . Non-small-cell lung cancer. Nat Rev Dis Primers. (2024) 10:71. doi: 10.1038/s41572-024-00551-9, 39327441

[3] Bray F Laversanne M Sung H Ferlay J Siegel RL Soerjomataram I . Global cancer statistics 2022: GLOBOCAN estimates of incidence and mortality worldwide for 36 cancers in 185 countries. CA Cancer J Clin. (2024) 74:229–63. doi: 10.3322/caac.21834, 38572751

[4] Chiou LJ Lin YY Lang HC. Effects of symptom burden on quality of life in patients with lung cancer. Curr Oncol. (2024) 31:6144–54. doi: 10.3390/curroncol31100458, 39451762 PMC11506357

[5] Yousefi M Jalilian H Heydari S Seyednejad F Mir N. Cost of lung cancer: a systematic review. Value Health Reg Issues. (2023) 33:17–26. doi: 10.1016/j.vhri.2022.07.007, 36201970

[6] Riely GJ Wood DE Ettinger DS Aisner DL Akerley W Bauman JR . Non-small cell lung cancer, version 4.2024, NCCN clinical practice guidelines in oncology. J Natl Compr Cancer Netw. (2024) 22:249–74. doi: 10.6004/jnccn.2204.0023 38754467

[7] Liu D Hu X Xiao C Bai J Barandouzi ZA Lee S . Evaluation of large language models in tailoring educational content for Cancer survivors and their caregivers: quality analysis. JMIR Cancer. (2025) 11:e67914. doi: 10.2196/67914, 40192716 PMC11995809

[8] Sallam M. ChatGPT utility in healthcare education, research, and practice: systematic review on the promising perspectives and valid concerns. Healthcare (Basel). (2023) 11:887. doi: 10.3390/healthcare11060887, 36981544 PMC10048148

[9] van Dis EAM Bollen J Zuidema W van Rooij R Bockting CL. ChatGPT: five priorities for research. Nature. (2023) 614:224–6. doi: 10.1038/d41586-023-00288-7, 36737653

[10] Bryson X Albarran M Pham N Salunga A Johnson T Hogue GD . Artificial intelligence-based large language models can facilitate patient education. J Pediatr Soc North Am. (2025) 12:100196. doi: 10.1016/j.jposna.2025.100196, 40791971 PMC12337203

[11] Ray PP. Servicing open-source large language models for oncology. Oncologist. (2025) 30:oyae264. doi: 10.1093/oncolo/oyae264, 39316690 PMC12395138

[12] Will J Gupta M Zaretsky J Dowlath A Testa P Feldman J. Enhancing the readability of online patient education materials using large language models: cross-sectional study. J Med Internet Res. (2025) 27:e69955. doi: 10.2196/69955, 40465378 PMC12177420

[13] Rodler S Cei F Ganjavi C Checcucci E De Backer P Rivero Belenchon I . GPT-4 generates accurate and readable patient education materials aligned with current oncological guidelines: a randomized assessment. PLoS One. (2025) 20:e0324175. doi: 10.1371/journal.pone.0324175, 40465696 PMC12136319

[14] CHART Collaborative. Reporting guideline for chatbot health advice studies: chatbot assessment reporting tool (CHART) statement. Ann Fam Med. (2025) 23:389–98. doi: 10.1370/afm.250386 40750305 PMC12459699

[15] Reuss JE Kuruvilla S Ismaila N Azar IH Feldman J Furuya N . Therapy for stage IV non-small cell lung Cancer with driver alterations: ASCO living guideline, version 2025.1. J Clin Oncol. (2025) 43:e31–44. doi: 10.1200/JCO-25-01061, 40674661

[16] Owen DH Halmos B Puri S Qin A Ismaila N Abu Rous F . Therapy for stage IV non-small cell lung Cancer without driver alterations: ASCO living guideline, version 2025.1. J Clin Oncol. (2025) 43:e45–58. doi: 10.1200/JCO-25-01062, 40674687

[17] Hendriks LE Kerr KM Menis J Mok TS Nestle U Passaro A . Non-oncogene-addicted metastatic non-small-cell lung cancer: ESMO clinical practice guideline for diagnosis, treatment and follow-up. Ann Oncol. (2023) 34:358–76. doi: 10.1016/j.annonc.2022.12.013, 36669645

[18] Charnock D Shepperd S Needham G Gann R. DISCERN: an instrument for judging the quality of written consumer health information on treatment choices. J Epidemiol Community Health. (1999) 53:105–11. doi: 10.1136/jech.53.2.105, 10396471 PMC1756830

[19] Hu M Zou P Li T Wang Y. Evaluating the quality of ChatGPT-generated medical information on major ophthalmic conditions: a comparative assessment against the EQIP tool and guidelines. PLoS One. (2025) 20:e0334250. doi: 10.1371/journal.pone.0334250, 41100573 PMC12530511

[20] Silberg WM Lundberg GD Musacchio RA. Assessing, controlling, and assuring the quality of medical information on the internet: Caveant lector et viewor—let the reader and viewer beware. JAMA. (1997) 277:1244–5. 9103351

[21] Ostrowska M Kacała P Onolememen D Vaughan-Lane K Sisily Joseph A Ostrowski A . To trust or not to trust: evaluating the reliability and safety of AI responses to laryngeal cancer queries. Eur Arch Otorrinolaringol. (2024) 281:6069–81. doi: 10.1007/s00405-024-08643-8, 38652298 PMC11512842

[22] Smith EA Senter RJ. Automated Readability Index. AMRL-TR-66-220.Wright-Patterson Air Force Base, OH, USA: Aerospace Medical Research Laboratories (1967). 5302480

[23] Coleman M Liau TL. A computer readability formula designed for machine scoring. J Appl Psychol. (1975) 60:283–4. doi: 10.1037/h0076540

[24] Kincaid JP Fishburne RP Jr Rogers RL Chissom BS. Derivation of New Readability Formulas for Navy Enlisted Personnel. Research Branch Report 8–75. Millington: Naval Technical Training Command (1975).

[25] Gunning R. The Technique of Clear Writing. New York: McGraw-Hill (1952).

[26] McLaughlin GH. Smog grading: a new readability formula. J Read. (1969) 12:639–46.

[27] Flesch R. A new readability yardstick. J Appl Psychol. (1948) 32:221–33. doi: 10.1037/h0057532, 18867058

[28] IBM Corp. IBM SPSS Statistics for Windows, Version 30.0. Armonk: IBM Corp (2024).

[29] OpenAI. Introducing GPT-5.4. Available online at: https://openai.com/index/introducing-gpt-5-4/ (accessed May 22, 2026).

[30] Google. Gemini 2.5: our most intelligent AI model. Google Blog. Available online at: https://blog.google/innovation-and-ai/models-and-research/google-deepmind/gemini-model-thinking-updates-march-2025/ (accessed May 22, 2026).

[31] DeepSeek. DeepSeek-V3-0324 Release. Available online at: https://api-docs.deepseek.com/news/news250325 (accessed May 22, 2026).

[32] Microsoft. Overview of Microsoft 365 Copilot Chat. Microsoft Learn. Available online at: https://learn.microsoft.com/en-us/copilot/overview (accessed May 22, 2026).

[33] ByteDance. Doubao: AI intelligent assistant. Available online at: https://www.doubao.com/ (accessed May 22, 2026).

