# Supplementary material for: Safety and quality of public chatbots for lung cancer prognostic information: a comparative evaluation
Source: Front Public Health. 2026 Aug 24;14:1935121. doi: 10.3389/fpubh.2026.1935121 (PMC13547269; doi:10.3389/fpubh.2026.1935121)
Supplement: Supplementary file 1 [file Table__1.docx]

Supplementary Material 1. Completed CHART Reporting Checklist

**Description: This file provides the completed CHART reporting checklist and maps each checklist item to the corresponding manuscript section.**

| **Heading** | **No** | **CHART checklist item** | **Page No** |
| --- | --- | --- | --- |
| **Title and abstract** |  |  | 1-2 |
| Title | 1a | State that the study is assessing one or more generative AI-driven chatbots for clinical evidence or health advice. | 1 |
| Abstract/summary | 1b | Apply a structured format, if applicable. | 1-2 |
| **Introduction** |  |  | 2-3 |
| Background | 2a | State the scientific background, rationale, and healthcare context for evaluating the generative AI-driven chatbot(s), referencing relevant literature when applicable. | 2-3 |
|  | 2b | State the aims and research questions including the target audience, intervention, comparator(s), and outcome(s). | 3 |
| **Methods** |  |  | 3-10 |
| Model identifiers | 3a | State the name and version identifier(s) of the generative AI model(s) and chatbot(s) under evaluation, as well as their date of release or last update. | 5; 22-25 |
|  | 3b | State whether the generative AI model(s) and chatbot(s) are open-source or closed-source/proprietary. | 5 |
| Model details | 4a | State whether the generative AI model was a base model or a novel base model, tuned model, or fine-tuned model. | 5-6* |
|  | 4b | If a base model is used, cite its development in sufficient detail to identify the model. | 5-6; 22-25 |
|  | 4c | If a novel base model, tuned model, or fine-tuned model is used, describe the pre- and/or post-implementation/deployment data and parameters. | 5-6† |
| Prompt engineering | 5a | Describe the evolution of study prompt development. | 6 |
|  | 5ai | Describe the sources of prompts. | 6 |
|  | 5aii | State the number and characteristics of the individual(s) involved in prompt engineering. | 6 |
|  | 5aiii | Provide details of any patient and public involvement during prompt engineering. | 6; 20 |
|  | 5b | Provide study prompts. | 6; Supplementary Material 2 |
| Query strategy | 6a | State route of access to generative AI model. | 5; 7 |
|  | 6b | State the date(s) and location(s) of queries for the generative AI-driven chatbot(s) including the day, month, and year as well as city and country. | 5 |
|  | 6c | Describe whether prompts were input into separate chat session(s). | 7 |
|  | 6d | Provide all generative AI-driven chatbot output/responses | 7; Supplementary Material 4 |
| Performance evaluation | 7a | Define the ground truth or reference standard used to define successful generative AI-driven chatbot performance. | 8; Supplementary Material 6 |
|  | 7b | Describe the process undertaken for generative AI-driven chatbot performance evaluation. | 8-9 |
|  | 7bi | State the number and characteristics of team members involved in performance evaluation. | 9; Supplementary Material 7 |
|  | 7bii | Provide details of any patients and public involvement during the evaluation process. | 9; 20 |
|  | 7biii | State whether evaluators were blinded to the identity of the generative AI-driven chatbot(s) under assessment. | 9 |
| Sample size | 8 | Report how the sample size was determined. | 6-7; 10‡ |
| Data analysis | 9a | Describe statistical analysis methods, including any evaluation of reproducibility of generative AI-driven chatbot responses. | 9-10; 18-19 |
|  | 9ai | Report the measures used for performance evaluation. | 8-10 |
| **Results** |  |  | 10-16 |
|  | 10a | Report the performance evaluation undertaken including the alignment between generative AI-driven chatbot output and ground truth or reference standard using quantitative or mixed methods approaches as applicable. | 10-15; Supplementary Material 6 |
|  | 10b | For responses deviating from the ground truth or reference standard, state the nature of the difference(s). | 15-16; Supplementary Material 6; Supplementary Material 9 |
|  | 10c | Report the evaluation for potentially harmful, biased, or misleading responses. | 16; Supplementary Material 8; Supplementary Material 9 |
| **Discussion** |  |  | 16-20 |
|  | 11a | Interpret study findings in the context of relevant evidence. | 16-18 |
|  | 11b | Describe the strengths and limitations of the study. | 18-19 |
|  | 11c | Describe the potential implications for practice, education, policy, regulation, and research. | 18-20 |
| **Open science** |  |  | 20-21 |
| Disclosures | 12a | Report any relevant conflicts of interest for all authors. | 20 |
| Funding | 12b | Report sources of funding and their role in the conduct and reporting of the study. | 20 |
| Ethics | 12c | Describe the process undertaken for ethical approval. | 20 |
|  | 12ci | Describe the measures taken to safeguard data privacy of patient health information, as applicable. | 20-21 |
|  | 12cii | State whether permission/licensing was obtained for the use of original, copyrighted data. | 21 |
| Protocol | 12d | Provide a study protocol. | 21§ |
| Data availability | 12e | State where study data, code repository, and model parameters can be accessed. | 21¶ |

Notes: Supplementary Material, supplementary file; AI, artificial intelligence; LLM, large language model; CHART, Chatbot Health Advice Reporting Transparency. * The manuscript evaluates public product-level systems; fixed backend base models were not independently auditable. † Proprietary deployment data, system prompts, routing mechanisms, and parameters were not externally accessible. ‡ Prompt-set size was determined by screening, domain coverage, and expert review; no formal power calculation was applicable. § The protocol was not prospectively registered; the prespecified prompt set, scoring rules, and analysis plan are described. ¶ Model parameters were not accessible; study materials and supplementary analyses are provided for transparency.
