# Supplementary material for: Safety and quality of public chatbots for lung cancer prognostic information: a comparative evaluation
Source: Front Public Health. 2026 Aug 24;14:1935121. doi: 10.3389/fpubh.2026.1935121 (PMC13547269; doi:10.3389/fpubh.2026.1935121)
Supplement: Supplementary file 3 [file Table__3.docx]

Supplementary Material 3. Question Inclusion/Exclusion Process and Search-Source Audit

**Description: This file provides the record of question inclusion and exclusion, together with the search-source audit used to construct the standardized lung cancer prognosis prompt bank.**

Optimization of a 100-item lung cancer prognosis question pool into a final 53-item patient-oriented English question bank

# Purpose

This document records the screening process used to select 53 patient-oriented and clinically relevant lung cancer prognosis questions from an original 100-item question pool for evaluating the reliability, readability, empathy, and safety of large language model responses.

Transparency Note

Publicly accessible search trend tools, online forums, question-answer platforms, patient communities, professional patient education materials, clinical guidelines, and thoracic oncology expert input were used to inform topic coverage and patient-facing wording. Exact platform-specific heat values, ranking values, and frequency counts were not prospectively archived. Therefore, the final question bank should be interpreted as a patient-oriented, clinically relevant, and expert-reviewed prompt set rather than a formal search-volume ranking.

# Screening Criteria

Direct relevance to lung cancer prognosis.

Patient-oriented relevance, direct prognostic relevance, broad topic coverage, and patient-facing wording.

Non-duplication across survival, stage, treatment, recurrence, metastasis, lifestyle, and decision-making topics.

Suitability for standardized single-turn large language model evaluation.

Avoidance of overly narrow, region-specific, ambiguous, or non-core prognosis topics.

# Selection Flow Summary

| **Step** | **Number of Questions** | **Action** | **Description** |
| --- | --- | --- | --- |
| Initial pool | 100 | Screened | Original patient-oriented questions across five domains. |
| Included | 53 | Retained | Final patient-oriented and clinically relevant questions retained for the formal question bank. |
| Excluded | 47 | Removed or merged | Questions excluded because of duplication, lower incremental contribution to topic coverage, indirect relevance, or narrow clinical scope. |
| Final set | 53 | Prepared for analysis | Questions used for LLM reliability and readability evaluation. |

# Domain-Level Screening Summary

| **Domain** | **Original Questions** | **Included** | **Excluded** |
| --- | --- | --- | --- |
| Survival Time and Survival Rates | 20 | 12 | 8 |
| Stage and Pathological Type | 20 | 10 | 10 |
| Treatment and Post-Treatment Prognosis | 20 | 12 | 8 |
| Recurrence, Metastasis, and Disease Progression | 20 | 11 | 9 |
| Lifestyle, Psychological Concerns, and Decision-Making | 20 | 8 | 12 |

# Full Inclusion/Exclusion Record Table

The table below documents the decision for each original question. Included questions are mapped to the final 53-item question bank; excluded questions include the primary rationale and retained/merged target where applicable.

| **Original No.** | **Original Domain** | **Original Question (English)** | **Decision** | **Final No.** | **Reason Category** | **Rationale for Inclusion or Exclusion** | **Retained / Merged Into** |
| --- | --- | --- | --- | --- | --- | --- | --- |
| 1 | Survival Time and Survival Rates | How long can a person with lung cancer usually live? | Included | 1 | Included | Retained for direct prognostic relevance, patient-oriented wording, broad topic coverage, and suitability for standardized single-turn LLM evaluation. | Final Q1 |
| 2 | Survival Time and Survival Rates | What is the 5-year survival rate for lung cancer? | Included | 2 | Included | Retained for direct prognostic relevance, patient-oriented wording, broad topic coverage, and suitability for standardized single-turn LLM evaluation. | Final Q2 |
| 3 | Survival Time and Survival Rates | How long can patients with stage I lung cancer usually live? | Included | 3 | Included | Retained for direct prognostic relevance, patient-oriented wording, broad topic coverage, and suitability for standardized single-turn LLM evaluation. | Final Q3 |
| 4 | Survival Time and Survival Rates | What is the prognosis of patients with stage II lung cancer? | Excluded |  | Merged Exclusion | Highly overlaps with Q3, Q5, and Q17 on stage-specific prognosis; stage II prognosis has lower incremental contribution to core topic coverage than stage I or stage IV prognosis. | Retained as Final Q3 and Final Q17 |
| 5 | Survival Time and Survival Rates | What is the average life expectancy for patients with stage III lung cancer? | Excluded |  | Merged Exclusion | Overlaps with Final Q17 on survival differences across stages and with other stage-specific life expectancy questions; stage III alone has lower search interest than stage IV. | Retained as Final Q17 |
| 6 | Survival Time and Survival Rates | How many years can patients with stage IV lung cancer live? | Included | 4 | Included | Retained for direct prognostic relevance, patient-oriented wording, broad topic coverage, and suitability for standardized single-turn LLM evaluation. | Final Q4 |
| 7 | Survival Time and Survival Rates | What is the longest survival time reported for advanced lung cancer patients? | Included | 5 | Included | Retained for direct prognostic relevance, patient-oriented wording, broad topic coverage, and suitability for standardized single-turn LLM evaluation. | Final Q5 |
| 8 | Survival Time and Survival Rates | How long do patients with small cell lung cancer usually live? | Included | 6 | Included | Retained for direct prognostic relevance, patient-oriented wording, broad topic coverage, and suitability for standardized single-turn LLM evaluation. | Final Q6 |
| 9 | Survival Time and Survival Rates | Is the survival rate of non-small cell lung cancer high? | Included | 7 | Included | Retained for direct prognostic relevance, patient-oriented wording, broad topic coverage, and suitability for standardized single-turn LLM evaluation. | Final Q7 |
| 10 | Survival Time and Survival Rates | How long can someone live with lung cancer without treatment? | Included | 8 | Included | Retained for direct prognostic relevance, patient-oriented wording, broad topic coverage, and suitability for standardized single-turn LLM evaluation. | Final Q8 |
| 11 | Survival Time and Survival Rates | How much can treatment usually extend survival time? | Included | 9 | Included | Retained for direct prognostic relevance, patient-oriented wording, broad topic coverage, and suitability for standardized single-turn LLM evaluation. | Final Q9 |
| 12 | Survival Time and Survival Rates | After lung cancer is cured, can patients have the same life expectancy as people without lung cancer? | Excluded |  | Excluded | The phrasing about a normal life expectancy after cure is overly idealized and may introduce uncontrolled individual variability; it is covered by surgery and cure-related questions. | Retained as Final Q24 |
| 13 | Survival Time and Survival Rates | Do younger lung cancer patients have a better prognosis? | Included | 10 | Included | Retained for direct prognostic relevance, patient-oriented wording, broad topic coverage, and suitability for standardized single-turn LLM evaluation. | Final Q10 |
| 14 | Survival Time and Survival Rates | Do older lung cancer patients usually have a shorter life expectancy? | Excluded |  | Excluded | This older-age question mirrors Final Q10 on prognosis in younger patients and adds limited incremental information. | Retained as Final Q10 |
| 15 | Survival Time and Survival Rates | Do female lung cancer patients live longer than male patients? | Excluded |  | Excluded | Sex differences are an epidemiological subgroup issue with relatively lower incremental contribution to core prognostic topic coverage and are not among the core high-frequency patient questions. | None |
| 16 | Survival Time and Survival Rates | Do non-smokers with lung cancer have a better prognosis? | Included | 11 | Included | Retained for direct prognostic relevance, patient-oriented wording, broad topic coverage, and suitability for standardized single-turn LLM evaluation. | Final Q11 |
| 17 | Survival Time and Survival Rates | Can lung cancer patients survive for more than 10 years? | Excluded |  | Merged Exclusion | The long-term survival question overlaps with Final Q5 on the longest survival time; the retained question better reflects public search wording. | Retained as Final Q5 |
| 18 | Survival Time and Survival Rates | What is the longest survival record for patients with lung cancer? | Excluded |  | Merged Exclusion | Overlaps with Final Q5 on longest survival time, and 'longest record' may encourage responses based on extreme case reports. | Retained as Final Q5 |
| 19 | Survival Time and Survival Rates | Does early detection of lung cancer significantly improve survival? | Included | 12 | Included | Retained for direct prognostic relevance, patient-oriented wording, broad topic coverage, and suitability for standardized single-turn LLM evaluation. | Final Q12 |
| 20 | Survival Time and Survival Rates | How much individual variation is there in average survival time among lung cancer patients? | Excluded |  | Excluded | This question emphasizes statistical individual variation, is more academic in wording, and has relatively low patient-search interest; it is less suitable for a concise standardized bank. | None |
| 21 | Stage and Pathological Type | Is stage I lung cancer curable? | Included | 13 | Included | Retained for direct prognostic relevance, patient-oriented wording, broad topic coverage, and suitability for standardized single-turn LLM evaluation. | Final Q13 |
| 22 | Stage and Pathological Type | Is stage II lung cancer considered serious? | Included | 14 | Included | Retained for direct prognostic relevance, patient-oriented wording, broad topic coverage, and suitability for standardized single-turn LLM evaluation. | Final Q14 |
| 23 | Stage and Pathological Type | Is stage III lung cancer already considered advanced disease? | Included | 15 | Included | Retained for direct prognostic relevance, patient-oriented wording, broad topic coverage, and suitability for standardized single-turn LLM evaluation. | Final Q15 |
| 24 | Stage and Pathological Type | Is there still hope for treatment in stage IV lung cancer? | Included | 16 | Included | Retained for direct prognostic relevance, patient-oriented wording, broad topic coverage, and suitability for standardized single-turn LLM evaluation. | Final Q16 |
| 25 | Stage and Pathological Type | Are survival rates very different across lung cancer stages? | Included | 17 | Included | Retained for direct prognostic relevance, patient-oriented wording, broad topic coverage, and suitability for standardized single-turn LLM evaluation. | Final Q17 |
| 26 | Stage and Pathological Type | What is the prognosis of lung cancer with brain metastasis? | Included | 18 | Included | Retained for direct prognostic relevance, patient-oriented wording, broad topic coverage, and suitability for standardized single-turn LLM evaluation. | Final Q18 |
| 27 | Stage and Pathological Type | How long can patients with bone metastases from lung cancer live? | Included | 19 | Included | Retained for direct prognostic relevance, patient-oriented wording, broad topic coverage, and suitability for standardized single-turn LLM evaluation. | Final Q19 |
| 28 | Stage and Pathological Type | What is the usual prognosis after liver metastasis from lung cancer? | Excluded |  | Excluded | Liver metastasis is clinically important, but brain and bone metastases are more central to patient-facing prognosis discussions and were prioritized in the 53-item set. | Retained as Final Q18 and Final Q19 |
| 29 | Stage and Pathological Type | Does pleural metastasis from lung cancer indicate a poorer prognosis? | Excluded |  | Excluded | Pleural metastasis or pleural disease is more specialized and has narrower patient-facing topic relevance than brain or bone metastasis. | Retained as Final Q18 and Final Q19 |
| 30 | Stage and Pathological Type | Does multiple metastatic lung cancer always mean a very poor prognosis? | Excluded |  | Merged Exclusion | Multiple metastases overlap with Final Q39 on whether metastatic lung cancer is no longer curable; the broader, higher-frequency wording was retained. | Retained as Final Q39 |
| 31 | Stage and Pathological Type | Why is small cell lung cancer usually associated with a poorer prognosis? | Included | 20 | Included | Retained for direct prognostic relevance, patient-oriented wording, broad topic coverage, and suitability for standardized single-turn LLM evaluation. | Final Q20 |
| 32 | Stage and Pathological Type | Is non-small cell lung cancer always better than small cell lung cancer? | Included | 21 | Included | Retained for direct prognostic relevance, patient-oriented wording, broad topic coverage, and suitability for standardized single-turn LLM evaluation. | Final Q21 |
| 33 | Stage and Pathological Type | What is the usual prognosis of lung adenocarcinoma? | Excluded |  | Excluded | Adenocarcinoma prognosis is relatively specialized and is indirectly covered by non-small cell lung cancer and EGFR/ALK molecular-subtype questions. | Retained as Final Q7, Final Q30, and Final Q31 |
| 34 | Stage and Pathological Type | What is the survival rate of lung squamous cell carcinoma? | Excluded |  | Excluded | Squamous cell carcinoma as a separate subtype has relatively lower search interest and can be covered under overall non-small cell lung cancer prognosis. | Retained as Final Q7 |
| 35 | Stage and Pathological Type | Is large cell lung cancer more dangerous? | Excluded |  | Excluded | Large cell lung cancer is relatively uncommon and has lower patient-facing topic relevance; it is not suitable for the core shortened question set. | None |
| 36 | Stage and Pathological Type | Does a higher lung cancer stage always mean shorter survival? | Excluded |  | Merged Exclusion | Overlaps with Final Q17 on survival differences across stages; Final Q17 is more neutral and better suited for model evaluation. | Retained as Final Q17 |
| 37 | Stage and Pathological Type | Is there still a chance of cure for locally advanced lung cancer? | Excluded |  | Merged Exclusion | Locally advanced disease overlaps with stage III/stage IV treatment-hope questions and is more difficult for lay users to define. | Retained as Final Q15 and Final Q16 |
| 38 | Stage and Pathological Type | Does lung cancer limited to the lung usually have a good prognosis? | Excluded |  | Merged Exclusion | Localized lung lesions overlap with questions on stage I curability and early detection. | Retained as Final Q12 and Final Q13 |
| 39 | Stage and Pathological Type | Does lymph node metastasis significantly worsen prognosis? | Included | 22 | Included | Retained for direct prognostic relevance, patient-oriented wording, broad topic coverage, and suitability for standardized single-turn LLM evaluation. | Final Q22 |
| 40 | Stage and Pathological Type | How much does pathological type affect lung cancer prognosis? | Excluded |  | Merged Exclusion | The overall effect of pathological type was decomposed into small cell versus non-small cell lung cancer questions, which are more specific and high-frequency. | Retained as Final Q20 and Final Q21 |
| 41 | Treatment and Post-Treatment Prognosis | How long can patients usually live after lung cancer surgery? | Included | 23 | Included | Retained for direct prognostic relevance, patient-oriented wording, broad topic coverage, and suitability for standardized single-turn LLM evaluation. | Final Q23 |
| 42 | Treatment and Post-Treatment Prognosis | Is surgical removal considered a cure for lung cancer? | Included | 24 | Included | Retained for direct prognostic relevance, patient-oriented wording, broad topic coverage, and suitability for standardized single-turn LLM evaluation. | Final Q24 |
| 43 | Treatment and Post-Treatment Prognosis | Is the recurrence rate high after lung cancer surgery? | Included | 25 | Included | Retained for direct prognostic relevance, patient-oriented wording, broad topic coverage, and suitability for standardized single-turn LLM evaluation. | Final Q25 |
| 44 | Treatment and Post-Treatment Prognosis | Can chemotherapy significantly extend survival in lung cancer patients? | Included | 26 | Included | Retained for direct prognostic relevance, patient-oriented wording, broad topic coverage, and suitability for standardized single-turn LLM evaluation. | Final Q26 |
| 45 | Treatment and Post-Treatment Prognosis | Does radiotherapy improve prognosis in lung cancer patients? | Included | 27 | Included | Retained for direct prognostic relevance, patient-oriented wording, broad topic coverage, and suitability for standardized single-turn LLM evaluation. | Final Q27 |
| 46 | Treatment and Post-Treatment Prognosis | Can immunotherapy improve survival rates in lung cancer? | Included | 28 | Included | Retained for direct prognostic relevance, patient-oriented wording, broad topic coverage, and suitability for standardized single-turn LLM evaluation. | Final Q28 |
| 47 | Treatment and Post-Treatment Prognosis | Can patients receiving targeted therapy live for many years? | Included | 29 | Included | Retained for direct prognostic relevance, patient-oriented wording, broad topic coverage, and suitability for standardized single-turn LLM evaluation. | Final Q29 |
| 48 | Treatment and Post-Treatment Prognosis | Do patients with EGFR-mutated lung cancer usually have a better prognosis? | Included | 30 | Included | Retained for direct prognostic relevance, patient-oriented wording, broad topic coverage, and suitability for standardized single-turn LLM evaluation. | Final Q30 |
| 49 | Treatment and Post-Treatment Prognosis | Do ALK-positive lung cancer patients have a good chance of long-term survival? | Included | 31 | Included | Retained for direct prognostic relevance, patient-oriented wording, broad topic coverage, and suitability for standardized single-turn LLM evaluation. | Final Q31 |
| 50 | Treatment and Post-Treatment Prognosis | Does high PD-L1 expression suggest a better treatment response? | Excluded |  | Excluded | PD-L1 expression is mainly a predictor of response to immunotherapy rather than a general public prognosis question; it is also relatively specialized. | Retained as Final Q28 |
| 51 | Treatment and Post-Treatment Prognosis | Does tumor shrinkage mean the prognosis has improved? | Excluded |  | Excluded | Tumor shrinkage is a treatment-response detail and overlaps with complete remission and post-treatment prognosis questions. | Retained as Final Q32 |
| 52 | Treatment and Post-Treatment Prognosis | Can lung cancer still recur after complete remission? | Included | 32 | Included | Retained for direct prognostic relevance, patient-oriented wording, broad topic coverage, and suitability for standardized single-turn LLM evaluation. | Final Q32 |
| 53 | Treatment and Post-Treatment Prognosis | Is combination therapy better than a single treatment? | Excluded |  | Excluded | Combination therapy is broad and highly context-dependent across guidelines; it has lower incremental contribution to core topic coverage than immunotherapy, targeted therapy, and surgery. | Retained as Final Q28 and Final Q29 |
| 54 | Treatment and Post-Treatment Prognosis | What options can extend survival after treatment failure? | Excluded |  | Merged Exclusion | Options after treatment failure overlap with questions on recurrence and treatment opportunities after multiple recurrences. | Retained as Final Q41 |
| 55 | Treatment and Post-Treatment Prognosis | Can clinical trial treatments improve lung cancer prognosis? | Excluded |  | Excluded | Clinical trials are important, but public search interest is relatively lower and access varies greatly across regions. | None |
| 56 | Treatment and Post-Treatment Prognosis | Can adjuvant therapy after surgery reduce the risk of recurrence? | Excluded |  | Merged Exclusion | Adjuvant therapy after surgery overlaps with postoperative recurrence risk, early treatment, and post-treatment prognosis. | Retained as Final Q25 and Final Q33 |
| 57 | Treatment and Post-Treatment Prognosis | Can side effects from chemoradiotherapy affect survival? | Excluded |  | Excluded | Side effects and survival relate more to treatment safety and tolerability than to core prognosis-search topics. | None |
| 58 | Treatment and Post-Treatment Prognosis | Does treatment interruption affect prognosis? | Excluded |  | Excluded | The effect of treatment interruption depends heavily on the individual treatment plan and has lower search interest. | None |
| 59 | Treatment and Post-Treatment Prognosis | Does starting treatment earlier lead to a better prognosis? | Included | 33 | Included | Retained for direct prognostic relevance, patient-oriented wording, broad topic coverage, and suitability for standardized single-turn LLM evaluation. | Final Q33 |
| 60 | Treatment and Post-Treatment Prognosis | Does precision medicine significantly improve lung cancer prognosis? | Included | 34 | Included | Retained for direct prognostic relevance, patient-oriented wording, broad topic coverage, and suitability for standardized single-turn LLM evaluation. | Final Q34 |
| 61 | Recurrence, Metastasis, and Disease Progression | When is lung cancer most likely to recur after treatment? | Included | 35 | Included | Retained for direct prognostic relevance, patient-oriented wording, broad topic coverage, and suitability for standardized single-turn LLM evaluation. | Final Q35 |
| 62 | Recurrence, Metastasis, and Disease Progression | How long can someone live after lung cancer recurrence? | Included | 36 | Included | Retained for direct prognostic relevance, patient-oriented wording, broad topic coverage, and suitability for standardized single-turn LLM evaluation. | Final Q36 |
| 63 | Recurrence, Metastasis, and Disease Progression | How many years without recurrence after surgery is considered relatively safe? | Included | 37 | Included | Retained for direct prognostic relevance, patient-oriented wording, broad topic coverage, and suitability for standardized single-turn LLM evaluation. | Final Q37 |
| 64 | Recurrence, Metastasis, and Disease Progression | Which is more serious: local recurrence or distant metastasis? | Included | 38 | Included | Retained for direct prognostic relevance, patient-oriented wording, broad topic coverage, and suitability for standardized single-turn LLM evaluation. | Final Q38 |
| 65 | Recurrence, Metastasis, and Disease Progression | Does metastasis mean lung cancer is no longer curable? | Included | 39 | Included | Retained for direct prognostic relevance, patient-oriented wording, broad topic coverage, and suitability for standardized single-turn LLM evaluation. | Final Q39 |
| 66 | Recurrence, Metastasis, and Disease Progression | Is long-term survival still possible after brain metastasis? | Included | 40 | Included | Retained for direct prognostic relevance, patient-oriented wording, broad topic coverage, and suitability for standardized single-turn LLM evaluation. | Final Q40 |
| 67 | Recurrence, Metastasis, and Disease Progression | Does pain after bone metastasis indicate disease worsening? | Excluded |  | Merged Exclusion | Pain from bone metastasis overlaps with bone-metastasis prognosis and disease-progression symptoms; the more common bone-metastasis survival question was retained. | Retained as Final Q19 |
| 68 | Recurrence, Metastasis, and Disease Progression | Can lung cancer recurrence be treated with surgery again? | Excluded |  | Excluded | Repeat surgery after recurrence is a specialized treatment decision with a narrow applicable population and narrower generalizability across patient-facing contexts. | None |
| 69 | Recurrence, Metastasis, and Disease Progression | Are there still treatment options after multiple recurrences? | Included | 41 | Included | Retained for direct prognostic relevance, patient-oriented wording, broad topic coverage, and suitability for standardized single-turn LLM evaluation. | Final Q41 |
| 70 | Recurrence, Metastasis, and Disease Progression | Does lung cancer usually metastasize quickly? | Excluded |  | Excluded | Metastatic speed is broad and difficult to answer accurately; it may lead to overgeneralized responses. | None |
| 71 | Recurrence, Metastasis, and Disease Progression | Can targeted therapy still be used after recurrence? | Excluded |  | Excluded | Use of targeted therapy after recurrence depends on mutation status and resistance mechanisms; it is too specialized for the core set and is better as an extended question. | None |
| 72 | Recurrence, Metastasis, and Disease Progression | What is the usual survival rate after lung cancer recurrence? | Excluded |  | Merged Exclusion | Overlaps with Final Q36 on survival after recurrence; the retained question better reflects patient search language. | Retained as Final Q36 |
| 73 | Recurrence, Metastasis, and Disease Progression | Does pleural effusion in lung cancer indicate a poor prognosis? | Excluded |  | Excluded | Pleural effusion is an important progression sign, but it has lower incremental contribution to core topic coverage and generalizability than coughing up blood, shortness of breath, and weight loss. | Retained as Final Q42, Final Q44, and Final Q45 |
| 74 | Recurrence, Metastasis, and Disease Progression | What are the common signs that lung cancer is getting worse? | Included | 42 | Included | Retained for direct prognostic relevance, patient-oriented wording, broad topic coverage, and suitability for standardized single-turn LLM evaluation. | Final Q42 |
| 75 | Recurrence, Metastasis, and Disease Progression | Does coughing up blood mean lung cancer has progressed? | Included | 43 | Included | Retained for direct prognostic relevance, patient-oriented wording, broad topic coverage, and suitability for standardized single-turn LLM evaluation. | Final Q43 |
| 76 | Recurrence, Metastasis, and Disease Progression | Does shortness of breath indicate severe disease progression? | Included | 44 | Included | Retained for direct prognostic relevance, patient-oriented wording, broad topic coverage, and suitability for standardized single-turn LLM evaluation. | Final Q44 |
| 77 | Recurrence, Metastasis, and Disease Progression | Does rapid weight loss suggest a poor prognosis? | Included | 45 | Included | Retained for direct prognostic relevance, patient-oriented wording, broad topic coverage, and suitability for standardized single-turn LLM evaluation. | Final Q45 |
| 78 | Recurrence, Metastasis, and Disease Progression | Is loss of appetite related to disease worsening? | Excluded |  | Merged Exclusion | Loss of appetite overlaps with weight loss/cachexia; rapid weight loss was retained because it has a stronger direct prognostic meaning. | Retained as Final Q45 |
| 79 | Recurrence, Metastasis, and Disease Progression | Do lung cancer patients without obvious symptoms have a better prognosis? | Excluded |  | Excluded | Prognosis in asymptomatic lung cancer involves screening, stage, and tumor burden; search interest is lower and it is already covered by early detection. | Retained as Final Q12 |
| 80 | Recurrence, Metastasis, and Disease Progression | What are the common signs near the end of life in advanced lung cancer? | Excluded |  | Excluded | End-of-life signs belong more to palliative care and symptom-management topics, carry high emotional burden, and are less central to reliability/readability evaluation of prognosis information. | None |
| 81 | Lifestyle, Psychological Concerns, and Decision-Making | How can lung cancer patients improve their survival chances? | Included | 46 | Included | Retained for direct prognostic relevance, patient-oriented wording, broad topic coverage, and suitability for standardized single-turn LLM evaluation. | Final Q46 |
| 82 | Lifestyle, Psychological Concerns, and Decision-Making | Does quitting smoking improve prognosis after lung cancer diagnosis? | Included | 47 | Included | Retained for direct prognostic relevance, patient-oriented wording, broad topic coverage, and suitability for standardized single-turn LLM evaluation. | Final Q47 |
| 83 | Lifestyle, Psychological Concerns, and Decision-Making | Can diet affect survival in lung cancer patients? | Included | 48 | Included | Retained for direct prognostic relevance, patient-oriented wording, broad topic coverage, and suitability for standardized single-turn LLM evaluation. | Final Q48 |
| 84 | Lifestyle, Psychological Concerns, and Decision-Making | Can exercise help extend survival time in lung cancer patients? | Included | 49 | Included | Retained for direct prognostic relevance, patient-oriented wording, broad topic coverage, and suitability for standardized single-turn LLM evaluation. | Final Q49 |
| 85 | Lifestyle, Psychological Concerns, and Decision-Making | Can maintaining a positive attitude really improve prognosis? | Included | 50 | Included | Retained for direct prognostic relevance, patient-oriented wording, broad topic coverage, and suitability for standardized single-turn LLM evaluation. | Final Q50 |
| 86 | Lifestyle, Psychological Concerns, and Decision-Making | Does sleep quality affect recovery from lung cancer? | Excluded |  | Excluded | Sleep quality is more related to recovery and quality of life and has a weaker direct connection with survival prognosis; it was not retained in the shortened set. | None |
| 87 | Lifestyle, Psychological Concerns, and Decision-Making | Are nutritional supplements helpful for lung cancer prognosis? | Excluded |  | Excluded | Nutritional supplements may introduce commercial and non-evidence-based content and overlap with the broader diet/nutrition question. | Retained as Final Q48 |
| 88 | Lifestyle, Psychological Concerns, and Decision-Making | Can traditional Chinese medicine improve lung cancer survival rates? | Excluded |  | Excluded | Traditional Chinese medicine involves regional cultural differences and heterogeneous evidence; it has narrower generalizability across patient-facing contexts and may deviate from the international research context. | None |
| 89 | Lifestyle, Psychological Concerns, and Decision-Making | How can nutritional status be improved after weight loss? | Excluded |  | Merged Exclusion | Nutritional management after weight loss overlaps with rapid weight loss as a prognostic sign and with diet-related questions. | Retained as Final Q45 and Final Q48 |
| 90 | Lifestyle, Psychological Concerns, and Decision-Making | How should lung cancer patients take care of themselves during recovery? | Excluded |  | Merged Exclusion | Recovery-phase self-care is overly broad and overlaps with smoking cessation, diet, and exercise questions. | Retained as Final Q47, Final Q48, and Final Q49 |
| 91 | Lifestyle, Psychological Concerns, and Decision-Making | Should lung cancer patients continue working? | Excluded |  | Excluded | Whether to continue working relates more to social functioning and occupational rehabilitation, with indirect prognosis relevance and lower incremental contribution to core topic coverage. | None |
| 92 | Lifestyle, Psychological Concerns, and Decision-Making | Can lung cancer patients travel normally? | Excluded |  | Excluded | Travel is mainly a lifestyle-arrangement issue rather than a core lung cancer prognosis-information topic. | None |
| 93 | Lifestyle, Psychological Concerns, and Decision-Making | How can lung cancer patients cope with anxiety about life expectancy? | Excluded |  | Merged Exclusion | Life-expectancy anxiety is already represented in questions about how long patients can live, hope in stage IV disease, and balancing survival time with quality of life. | Retained as Final Q1, Final Q16, and Final Q53 |
| 94 | Lifestyle, Psychological Concerns, and Decision-Making | How can family members help lung cancer patients improve quality of life? | Excluded |  | Excluded | Family support mainly concerns caregiving and quality of life rather than core evaluation of prognosis information reliability/readability. | None |
| 95 | Lifestyle, Psychological Concerns, and Decision-Making | Should patients proactively ask their doctors about prognosis and expected survival time? | Excluded |  | Excluded | Whether patients should ask doctors about prognosis time is a communication-strategy issue and only indirectly related to model-output quality evaluation. | None |
| 96 | Lifestyle, Psychological Concerns, and Decision-Making | Is it appropriate for lung cancer patients to use AI to obtain information? | Excluded |  | Excluded | Using AI to obtain information is part of the study context rather than lung cancer prognostic medical content itself. | None |
| 97 | Lifestyle, Psychological Concerns, and Decision-Making | Is all online information about lung cancer prognosis trustworthy? | Included | 51 | Included | Retained for direct prognostic relevance, patient-oriented wording, broad topic coverage, and suitability for standardized single-turn LLM evaluation. | Final Q51 |
| 98 | Lifestyle, Psychological Concerns, and Decision-Making | Who should patients trust when they receive conflicting information? | Included | 52 | Included | Retained for direct prognostic relevance, patient-oriented wording, broad topic coverage, and suitability for standardized single-turn LLM evaluation. | Final Q52 |
| 99 | Lifestyle, Psychological Concerns, and Decision-Making | What are the most important factors in treatment decision-making for lung cancer patients? | Excluded |  | Merged Exclusion | Key factors in treatment decision-making overlap with Final Q53 on balancing survival time and quality of life, which better captures prognosis and value trade-offs. | Retained as Final Q53 |
| 100 | Lifestyle, Psychological Concerns, and Decision-Making | How should patients balance survival time and quality of life? | Included | 53 | Included | Retained for direct prognostic relevance, patient-oriented wording, broad topic coverage, and suitability for standardized single-turn LLM evaluation. | Final Q53 |
