# Supplementary material for: Safety and quality of public chatbots for lung cancer prognostic information: a comparative evaluation
Source: Front Public Health. 2026 Aug 24;14:1935121. doi: 10.3389/fpubh.2026.1935121 (PMC13547269; doi:10.3389/fpubh.2026.1935121)
Supplement: Supplementary file 5 [file Table__5.docx]

Supplementary Material 5. Model-Question-Level Query Log

**Description: This file provides the model-question-level query log, including question number, chatbot model, query window, access route, resubmission status, and saved file name.**

Purpose. This supplementary material documents the model-question-level query log for the 265 chatbot responses analyzed in the study. Each row in the main table represents one standardized lung cancer prognosis prompt submitted to one publicly accessible chatbot system.

Transparency note. Exact model-question-level timestamps were not prospectively archived during the original data collection process. Therefore, exact query times are reported as unavailable rather than retrospectively reconstructed or imputed. The predefined query window was April 1-21, 2026. Resubmission status is reported as no documented resubmission when no resubmission event was recorded in the retained response files. The saved file names refer to the model-wise response records used as raw-output source files for the cleaned response record.

Access conditions. All models were accessed through their official publicly available web-based user interfaces without API assistance. Prompts were submitted in English using identical wording across models, with one response collected per model per question.

## Model-wise saved response file map

| **Model** | **Standardizedsavedfilenameusedinthislog** | **Originaluploaded/sourcefilename** |
| --- | --- | --- |
| ChatGPT | Raw_ChatGPT.docx | （英文）肺癌预后ChatGpt(5).docx |
| Gemini | Raw_Gemini.docx | （英文）肺癌预后Gemini(4).docx |
| Copilot | Raw_Copilot.docx | （英文）肺癌预后Copilot(3).docx |
| DeepSeek | Raw_DeepSeek.docx | （英文）肺癌预后deepseek(3).docx |
| Doubao | Raw_Doubao.docx | （英文）肺癌预后Doubao(3).docx |

## Variable definitions

| **Variable** | **Definition and coding** |
| --- | --- |
| question no. | Question identifier from Q1 to Q53, corresponding to the final standardized 53-item lung cancer prognosis question bank. |
| model | Chatbot system evaluated: ChatGPT, Gemini, Copilot, DeepSeek, or Doubao. |
| date | Predefined query window. Exact model-question-level dates were not prospectively archived; therefore, the query window is reported as April 1-21, 2026. |
| time | Exact query time. Reported as not prospectively archived when the exact time was not retained. |
| access route | Official publicly available web-based interface for the corresponding chatbot; no API assistance was used. |
| resubmission | Whether a query was repeated because of technical interruption. “No documented resubmission” means no resubmission event was recorded in the retained files. |
| saved file name | Model-wise saved response record containing the raw or retained model output used for the cleaned response record. |

## Model-question-level query log

| **question no.** | **model** | **date** | **time** | **access route** | **resubmission** | **saved file name** |
| --- | --- | --- | --- | --- | --- | --- |
| Q1 | ChatGPT | April 1-21, 2026 (query window) | Not prospectively archived | ChatGPT web (official; no API) | No documented resubmission | Raw_ChatGPT.docx |
| Q1 | Gemini | April 1-21, 2026 (query window) | Not prospectively archived | Gemini web (official; no API) | No documented resubmission | Raw_Gemini.docx |
| Q1 | Copilot | April 1-21, 2026 (query window) | Not prospectively archived | Microsoft Copilot web (official; no API) | No documented resubmission | Raw_Copilot.docx |
| Q1 | DeepSeek | April 1-21, 2026 (query window) | Not prospectively archived | DeepSeek web (official; no API) | No documented resubmission | Raw_DeepSeek.docx |
| Q1 | Doubao | April 1-21, 2026 (query window) | Not prospectively archived | Doubao web (official; no API) | No documented resubmission | Raw_Doubao.docx |
| Q2 | ChatGPT | April 1-21, 2026 (query window) | Not prospectively archived | ChatGPT web (official; no API) | No documented resubmission | Raw_ChatGPT.docx |
| Q2 | Gemini | April 1-21, 2026 (query window) | Not prospectively archived | Gemini web (official; no API) | No documented resubmission | Raw_Gemini.docx |
| Q2 | Copilot | April 1-21, 2026 (query window) | Not prospectively archived | Microsoft Copilot web (official; no API) | No documented resubmission | Raw_Copilot.docx |
| Q2 | DeepSeek | April 1-21, 2026 (query window) | Not prospectively archived | DeepSeek web (official; no API) | No documented resubmission | Raw_DeepSeek.docx |
| Q2 | Doubao | April 1-21, 2026 (query window) | Not prospectively archived | Doubao web (official; no API) | No documented resubmission | Raw_Doubao.docx |
| Q3 | ChatGPT | April 1-21, 2026 (query window) | Not prospectively archived | ChatGPT web (official; no API) | No documented resubmission | Raw_ChatGPT.docx |
| Q3 | Gemini | April 1-21, 2026 (query window) | Not prospectively archived | Gemini web (official; no API) | No documented resubmission | Raw_Gemini.docx |
| Q3 | Copilot | April 1-21, 2026 (query window) | Not prospectively archived | Microsoft Copilot web (official; no API) | No documented resubmission | Raw_Copilot.docx |
| Q3 | DeepSeek | April 1-21, 2026 (query window) | Not prospectively archived | DeepSeek web (official; no API) | No documented resubmission | Raw_DeepSeek.docx |
| Q3 | Doubao | April 1-21, 2026 (query window) | Not prospectively archived | Doubao web (official; no API) | No documented resubmission | Raw_Doubao.docx |
| Q4 | ChatGPT | April 1-21, 2026 (query window) | Not prospectively archived | ChatGPT web (official; no API) | No documented resubmission | Raw_ChatGPT.docx |
| Q4 | Gemini | April 1-21, 2026 (query window) | Not prospectively archived | Gemini web (official; no API) | No documented resubmission | Raw_Gemini.docx |
| Q4 | Copilot | April 1-21, 2026 (query window) | Not prospectively archived | Microsoft Copilot web (official; no API) | No documented resubmission | Raw_Copilot.docx |
| Q4 | DeepSeek | April 1-21, 2026 (query window) | Not prospectively archived | DeepSeek web (official; no API) | No documented resubmission | Raw_DeepSeek.docx |
| Q4 | Doubao | April 1-21, 2026 (query window) | Not prospectively archived | Doubao web (official; no API) | No documented resubmission | Raw_Doubao.docx |
| Q5 | ChatGPT | April 1-21, 2026 (query window) | Not prospectively archived | ChatGPT web (official; no API) | No documented resubmission | Raw_ChatGPT.docx |
| Q5 | Gemini | April 1-21, 2026 (query window) | Not prospectively archived | Gemini web (official; no API) | No documented resubmission | Raw_Gemini.docx |
| Q5 | Copilot | April 1-21, 2026 (query window) | Not prospectively archived | Microsoft Copilot web (official; no API) | No documented resubmission | Raw_Copilot.docx |
| Q5 | DeepSeek | April 1-21, 2026 (query window) | Not prospectively archived | DeepSeek web (official; no API) | No documented resubmission | Raw_DeepSeek.docx |
| Q5 | Doubao | April 1-21, 2026 (query window) | Not prospectively archived | Doubao web (official; no API) | No documented resubmission | Raw_Doubao.docx |
| Q6 | ChatGPT | April 1-21, 2026 (query window) | Not prospectively archived | ChatGPT web (official; no API) | No documented resubmission | Raw_ChatGPT.docx |
| Q6 | Gemini | April 1-21, 2026 (query window) | Not prospectively archived | Gemini web (official; no API) | No documented resubmission | Raw_Gemini.docx |
| Q6 | Copilot | April 1-21, 2026 (query window) | Not prospectively archived | Microsoft Copilot web (official; no API) | No documented resubmission | Raw_Copilot.docx |
| Q6 | DeepSeek | April 1-21, 2026 (query window) | Not prospectively archived | DeepSeek web (official; no API) | No documented resubmission | Raw_DeepSeek.docx |
| Q6 | Doubao | April 1-21, 2026 (query window) | Not prospectively archived | Doubao web (official; no API) | No documented resubmission | Raw_Doubao.docx |
| Q7 | ChatGPT | April 1-21, 2026 (query window) | Not prospectively archived | ChatGPT web (official; no API) | No documented resubmission | Raw_ChatGPT.docx |
| Q7 | Gemini | April 1-21, 2026 (query window) | Not prospectively archived | Gemini web (official; no API) | No documented resubmission | Raw_Gemini.docx |
| Q7 | Copilot | April 1-21, 2026 (query window) | Not prospectively archived | Microsoft Copilot web (official; no API) | No documented resubmission | Raw_Copilot.docx |
| Q7 | DeepSeek | April 1-21, 2026 (query window) | Not prospectively archived | DeepSeek web (official; no API) | No documented resubmission | Raw_DeepSeek.docx |
| Q7 | Doubao | April 1-21, 2026 (query window) | Not prospectively archived | Doubao web (official; no API) | No documented resubmission | Raw_Doubao.docx |
| Q8 | ChatGPT | April 1-21, 2026 (query window) | Not prospectively archived | ChatGPT web (official; no API) | No documented resubmission | Raw_ChatGPT.docx |
| Q8 | Gemini | April 1-21, 2026 (query window) | Not prospectively archived | Gemini web (official; no API) | No documented resubmission | Raw_Gemini.docx |
| Q8 | Copilot | April 1-21, 2026 (query window) | Not prospectively archived | Microsoft Copilot web (official; no API) | No documented resubmission | Raw_Copilot.docx |
| Q8 | DeepSeek | April 1-21, 2026 (query window) | Not prospectively archived | DeepSeek web (official; no API) | No documented resubmission | Raw_DeepSeek.docx |
| Q8 | Doubao | April 1-21, 2026 (query window) | Not prospectively archived | Doubao web (official; no API) | No documented resubmission | Raw_Doubao.docx |
| Q9 | ChatGPT | April 1-21, 2026 (query window) | Not prospectively archived | ChatGPT web (official; no API) | No documented resubmission | Raw_ChatGPT.docx |
| Q9 | Gemini | April 1-21, 2026 (query window) | Not prospectively archived | Gemini web (official; no API) | No documented resubmission | Raw_Gemini.docx |
| Q9 | Copilot | April 1-21, 2026 (query window) | Not prospectively archived | Microsoft Copilot web (official; no API) | No documented resubmission | Raw_Copilot.docx |
| Q9 | DeepSeek | April 1-21, 2026 (query window) | Not prospectively archived | DeepSeek web (official; no API) | No documented resubmission | Raw_DeepSeek.docx |
| Q9 | Doubao | April 1-21, 2026 (query window) | Not prospectively archived | Doubao web (official; no API) | No documented resubmission | Raw_Doubao.docx |
| Q10 | ChatGPT | April 1-21, 2026 (query window) | Not prospectively archived | ChatGPT web (official; no API) | No documented resubmission | Raw_ChatGPT.docx |
| Q10 | Gemini | April 1-21, 2026 (query window) | Not prospectively archived | Gemini web (official; no API) | No documented resubmission | Raw_Gemini.docx |
| Q10 | Copilot | April 1-21, 2026 (query window) | Not prospectively archived | Microsoft Copilot web (official; no API) | No documented resubmission | Raw_Copilot.docx |
| Q10 | DeepSeek | April 1-21, 2026 (query window) | Not prospectively archived | DeepSeek web (official; no API) | No documented resubmission | Raw_DeepSeek.docx |
| Q10 | Doubao | April 1-21, 2026 (query window) | Not prospectively archived | Doubao web (official; no API) | No documented resubmission | Raw_Doubao.docx |
| Q11 | ChatGPT | April 1-21, 2026 (query window) | Not prospectively archived | ChatGPT web (official; no API) | No documented resubmission | Raw_ChatGPT.docx |
| Q11 | Gemini | April 1-21, 2026 (query window) | Not prospectively archived | Gemini web (official; no API) | No documented resubmission | Raw_Gemini.docx |
| Q11 | Copilot | April 1-21, 2026 (query window) | Not prospectively archived | Microsoft Copilot web (official; no API) | No documented resubmission | Raw_Copilot.docx |
| Q11 | DeepSeek | April 1-21, 2026 (query window) | Not prospectively archived | DeepSeek web (official; no API) | No documented resubmission | Raw_DeepSeek.docx |
| Q11 | Doubao | April 1-21, 2026 (query window) | Not prospectively archived | Doubao web (official; no API) | No documented resubmission | Raw_Doubao.docx |
| Q12 | ChatGPT | April 1-21, 2026 (query window) | Not prospectively archived | ChatGPT web (official; no API) | No documented resubmission | Raw_ChatGPT.docx |
| Q12 | Gemini | April 1-21, 2026 (query window) | Not prospectively archived | Gemini web (official; no API) | No documented resubmission | Raw_Gemini.docx |
| Q12 | Copilot | April 1-21, 2026 (query window) | Not prospectively archived | Microsoft Copilot web (official; no API) | No documented resubmission | Raw_Copilot.docx |
| Q12 | DeepSeek | April 1-21, 2026 (query window) | Not prospectively archived | DeepSeek web (official; no API) | No documented resubmission | Raw_DeepSeek.docx |
| Q12 | Doubao | April 1-21, 2026 (query window) | Not prospectively archived | Doubao web (official; no API) | No documented resubmission | Raw_Doubao.docx |
| Q13 | ChatGPT | April 1-21, 2026 (query window) | Not prospectively archived | ChatGPT web (official; no API) | No documented resubmission | Raw_ChatGPT.docx |
| Q13 | Gemini | April 1-21, 2026 (query window) | Not prospectively archived | Gemini web (official; no API) | No documented resubmission | Raw_Gemini.docx |
| Q13 | Copilot | April 1-21, 2026 (query window) | Not prospectively archived | Microsoft Copilot web (official; no API) | No documented resubmission | Raw_Copilot.docx |
| Q13 | DeepSeek | April 1-21, 2026 (query window) | Not prospectively archived | DeepSeek web (official; no API) | No documented resubmission | Raw_DeepSeek.docx |
| Q13 | Doubao | April 1-21, 2026 (query window) | Not prospectively archived | Doubao web (official; no API) | No documented resubmission | Raw_Doubao.docx |
| Q14 | ChatGPT | April 1-21, 2026 (query window) | Not prospectively archived | ChatGPT web (official; no API) | No documented resubmission | Raw_ChatGPT.docx |
| Q14 | Gemini | April 1-21, 2026 (query window) | Not prospectively archived | Gemini web (official; no API) | No documented resubmission | Raw_Gemini.docx |
| Q14 | Copilot | April 1-21, 2026 (query window) | Not prospectively archived | Microsoft Copilot web (official; no API) | No documented resubmission | Raw_Copilot.docx |
| Q14 | DeepSeek | April 1-21, 2026 (query window) | Not prospectively archived | DeepSeek web (official; no API) | No documented resubmission | Raw_DeepSeek.docx |
| Q14 | Doubao | April 1-21, 2026 (query window) | Not prospectively archived | Doubao web (official; no API) | No documented resubmission | Raw_Doubao.docx |
| Q15 | ChatGPT | April 1-21, 2026 (query window) | Not prospectively archived | ChatGPT web (official; no API) | No documented resubmission | Raw_ChatGPT.docx |
| Q15 | Gemini | April 1-21, 2026 (query window) | Not prospectively archived | Gemini web (official; no API) | No documented resubmission | Raw_Gemini.docx |
| Q15 | Copilot | April 1-21, 2026 (query window) | Not prospectively archived | Microsoft Copilot web (official; no API) | No documented resubmission | Raw_Copilot.docx |
| Q15 | DeepSeek | April 1-21, 2026 (query window) | Not prospectively archived | DeepSeek web (official; no API) | No documented resubmission | Raw_DeepSeek.docx |
| Q15 | Doubao | April 1-21, 2026 (query window) | Not prospectively archived | Doubao web (official; no API) | No documented resubmission | Raw_Doubao.docx |
| Q16 | ChatGPT | April 1-21, 2026 (query window) | Not prospectively archived | ChatGPT web (official; no API) | No documented resubmission | Raw_ChatGPT.docx |
| Q16 | Gemini | April 1-21, 2026 (query window) | Not prospectively archived | Gemini web (official; no API) | No documented resubmission | Raw_Gemini.docx |
| Q16 | Copilot | April 1-21, 2026 (query window) | Not prospectively archived | Microsoft Copilot web (official; no API) | No documented resubmission | Raw_Copilot.docx |
| Q16 | DeepSeek | April 1-21, 2026 (query window) | Not prospectively archived | DeepSeek web (official; no API) | No documented resubmission | Raw_DeepSeek.docx |
| Q16 | Doubao | April 1-21, 2026 (query window) | Not prospectively archived | Doubao web (official; no API) | No documented resubmission | Raw_Doubao.docx |
| Q17 | ChatGPT | April 1-21, 2026 (query window) | Not prospectively archived | ChatGPT web (official; no API) | No documented resubmission | Raw_ChatGPT.docx |
| Q17 | Gemini | April 1-21, 2026 (query window) | Not prospectively archived | Gemini web (official; no API) | No documented resubmission | Raw_Gemini.docx |
| Q17 | Copilot | April 1-21, 2026 (query window) | Not prospectively archived | Microsoft Copilot web (official; no API) | No documented resubmission | Raw_Copilot.docx |
| Q17 | DeepSeek | April 1-21, 2026 (query window) | Not prospectively archived | DeepSeek web (official; no API) | No documented resubmission | Raw_DeepSeek.docx |
| Q17 | Doubao | April 1-21, 2026 (query window) | Not prospectively archived | Doubao web (official; no API) | No documented resubmission | Raw_Doubao.docx |
| Q18 | ChatGPT | April 1-21, 2026 (query window) | Not prospectively archived | ChatGPT web (official; no API) | No documented resubmission | Raw_ChatGPT.docx |
| Q18 | Gemini | April 1-21, 2026 (query window) | Not prospectively archived | Gemini web (official; no API) | No documented resubmission | Raw_Gemini.docx |
| Q18 | Copilot | April 1-21, 2026 (query window) | Not prospectively archived | Microsoft Copilot web (official; no API) | No documented resubmission | Raw_Copilot.docx |
| Q18 | DeepSeek | April 1-21, 2026 (query window) | Not prospectively archived | DeepSeek web (official; no API) | No documented resubmission | Raw_DeepSeek.docx |
| Q18 | Doubao | April 1-21, 2026 (query window) | Not prospectively archived | Doubao web (official; no API) | No documented resubmission | Raw_Doubao.docx |
| Q19 | ChatGPT | April 1-21, 2026 (query window) | Not prospectively archived | ChatGPT web (official; no API) | No documented resubmission | Raw_ChatGPT.docx |
| Q19 | Gemini | April 1-21, 2026 (query window) | Not prospectively archived | Gemini web (official; no API) | No documented resubmission | Raw_Gemini.docx |
| Q19 | Copilot | April 1-21, 2026 (query window) | Not prospectively archived | Microsoft Copilot web (official; no API) | No documented resubmission | Raw_Copilot.docx |
| Q19 | DeepSeek | April 1-21, 2026 (query window) | Not prospectively archived | DeepSeek web (official; no API) | No documented resubmission | Raw_DeepSeek.docx |
| Q19 | Doubao | April 1-21, 2026 (query window) | Not prospectively archived | Doubao web (official; no API) | No documented resubmission | Raw_Doubao.docx |
| Q20 | ChatGPT | April 1-21, 2026 (query window) | Not prospectively archived | ChatGPT web (official; no API) | No documented resubmission | Raw_ChatGPT.docx |
| Q20 | Gemini | April 1-21, 2026 (query window) | Not prospectively archived | Gemini web (official; no API) | No documented resubmission | Raw_Gemini.docx |
| Q20 | Copilot | April 1-21, 2026 (query window) | Not prospectively archived | Microsoft Copilot web (official; no API) | No documented resubmission | Raw_Copilot.docx |
| Q20 | DeepSeek | April 1-21, 2026 (query window) | Not prospectively archived | DeepSeek web (official; no API) | No documented resubmission | Raw_DeepSeek.docx |
| Q20 | Doubao | April 1-21, 2026 (query window) | Not prospectively archived | Doubao web (official; no API) | No documented resubmission | Raw_Doubao.docx |
| Q21 | ChatGPT | April 1-21, 2026 (query window) | Not prospectively archived | ChatGPT web (official; no API) | No documented resubmission | Raw_ChatGPT.docx |
| Q21 | Gemini | April 1-21, 2026 (query window) | Not prospectively archived | Gemini web (official; no API) | No documented resubmission | Raw_Gemini.docx |
| Q21 | Copilot | April 1-21, 2026 (query window) | Not prospectively archived | Microsoft Copilot web (official; no API) | No documented resubmission | Raw_Copilot.docx |
| Q21 | DeepSeek | April 1-21, 2026 (query window) | Not prospectively archived | DeepSeek web (official; no API) | No documented resubmission | Raw_DeepSeek.docx |
| Q21 | Doubao | April 1-21, 2026 (query window) | Not prospectively archived | Doubao web (official; no API) | No documented resubmission | Raw_Doubao.docx |
| Q22 | ChatGPT | April 1-21, 2026 (query window) | Not prospectively archived | ChatGPT web (official; no API) | No documented resubmission | Raw_ChatGPT.docx |
| Q22 | Gemini | April 1-21, 2026 (query window) | Not prospectively archived | Gemini web (official; no API) | No documented resubmission | Raw_Gemini.docx |
| Q22 | Copilot | April 1-21, 2026 (query window) | Not prospectively archived | Microsoft Copilot web (official; no API) | No documented resubmission | Raw_Copilot.docx |
| Q22 | DeepSeek | April 1-21, 2026 (query window) | Not prospectively archived | DeepSeek web (official; no API) | No documented resubmission | Raw_DeepSeek.docx |
| Q22 | Doubao | April 1-21, 2026 (query window) | Not prospectively archived | Doubao web (official; no API) | No documented resubmission | Raw_Doubao.docx |
| Q23 | ChatGPT | April 1-21, 2026 (query window) | Not prospectively archived | ChatGPT web (official; no API) | No documented resubmission | Raw_ChatGPT.docx |
| Q23 | Gemini | April 1-21, 2026 (query window) | Not prospectively archived | Gemini web (official; no API) | No documented resubmission | Raw_Gemini.docx |
| Q23 | Copilot | April 1-21, 2026 (query window) | Not prospectively archived | Microsoft Copilot web (official; no API) | No documented resubmission | Raw_Copilot.docx |
| Q23 | DeepSeek | April 1-21, 2026 (query window) | Not prospectively archived | DeepSeek web (official; no API) | No documented resubmission | Raw_DeepSeek.docx |
| Q23 | Doubao | April 1-21, 2026 (query window) | Not prospectively archived | Doubao web (official; no API) | No documented resubmission | Raw_Doubao.docx |
| Q24 | ChatGPT | April 1-21, 2026 (query window) | Not prospectively archived | ChatGPT web (official; no API) | No documented resubmission | Raw_ChatGPT.docx |
| Q24 | Gemini | April 1-21, 2026 (query window) | Not prospectively archived | Gemini web (official; no API) | No documented resubmission | Raw_Gemini.docx |
| Q24 | Copilot | April 1-21, 2026 (query window) | Not prospectively archived | Microsoft Copilot web (official; no API) | No documented resubmission | Raw_Copilot.docx |
| Q24 | DeepSeek | April 1-21, 2026 (query window) | Not prospectively archived | DeepSeek web (official; no API) | No documented resubmission | Raw_DeepSeek.docx |
| Q24 | Doubao | April 1-21, 2026 (query window) | Not prospectively archived | Doubao web (official; no API) | No documented resubmission | Raw_Doubao.docx |
| Q25 | ChatGPT | April 1-21, 2026 (query window) | Not prospectively archived | ChatGPT web (official; no API) | No documented resubmission | Raw_ChatGPT.docx |
| Q25 | Gemini | April 1-21, 2026 (query window) | Not prospectively archived | Gemini web (official; no API) | No documented resubmission | Raw_Gemini.docx |
| Q25 | Copilot | April 1-21, 2026 (query window) | Not prospectively archived | Microsoft Copilot web (official; no API) | No documented resubmission | Raw_Copilot.docx |
| Q25 | DeepSeek | April 1-21, 2026 (query window) | Not prospectively archived | DeepSeek web (official; no API) | No documented resubmission | Raw_DeepSeek.docx |
| Q25 | Doubao | April 1-21, 2026 (query window) | Not prospectively archived | Doubao web (official; no API) | No documented resubmission | Raw_Doubao.docx |
| Q26 | ChatGPT | April 1-21, 2026 (query window) | Not prospectively archived | ChatGPT web (official; no API) | No documented resubmission | Raw_ChatGPT.docx |
| Q26 | Gemini | April 1-21, 2026 (query window) | Not prospectively archived | Gemini web (official; no API) | No documented resubmission | Raw_Gemini.docx |
| Q26 | Copilot | April 1-21, 2026 (query window) | Not prospectively archived | Microsoft Copilot web (official; no API) | No documented resubmission | Raw_Copilot.docx |
| Q26 | DeepSeek | April 1-21, 2026 (query window) | Not prospectively archived | DeepSeek web (official; no API) | No documented resubmission | Raw_DeepSeek.docx |
| Q26 | Doubao | April 1-21, 2026 (query window) | Not prospectively archived | Doubao web (official; no API) | No documented resubmission | Raw_Doubao.docx |
| Q27 | ChatGPT | April 1-21, 2026 (query window) | Not prospectively archived | ChatGPT web (official; no API) | No documented resubmission | Raw_ChatGPT.docx |
| Q27 | Gemini | April 1-21, 2026 (query window) | Not prospectively archived | Gemini web (official; no API) | No documented resubmission | Raw_Gemini.docx |
| Q27 | Copilot | April 1-21, 2026 (query window) | Not prospectively archived | Microsoft Copilot web (official; no API) | No documented resubmission | Raw_Copilot.docx |
| Q27 | DeepSeek | April 1-21, 2026 (query window) | Not prospectively archived | DeepSeek web (official; no API) | No documented resubmission | Raw_DeepSeek.docx |
| Q27 | Doubao | April 1-21, 2026 (query window) | Not prospectively archived | Doubao web (official; no API) | No documented resubmission | Raw_Doubao.docx |
| Q28 | ChatGPT | April 1-21, 2026 (query window) | Not prospectively archived | ChatGPT web (official; no API) | No documented resubmission | Raw_ChatGPT.docx |
| Q28 | Gemini | April 1-21, 2026 (query window) | Not prospectively archived | Gemini web (official; no API) | No documented resubmission | Raw_Gemini.docx |
| Q28 | Copilot | April 1-21, 2026 (query window) | Not prospectively archived | Microsoft Copilot web (official; no API) | No documented resubmission | Raw_Copilot.docx |
| Q28 | DeepSeek | April 1-21, 2026 (query window) | Not prospectively archived | DeepSeek web (official; no API) | No documented resubmission | Raw_DeepSeek.docx |
| Q28 | Doubao | April 1-21, 2026 (query window) | Not prospectively archived | Doubao web (official; no API) | No documented resubmission | Raw_Doubao.docx |
| Q29 | ChatGPT | April 1-21, 2026 (query window) | Not prospectively archived | ChatGPT web (official; no API) | No documented resubmission | Raw_ChatGPT.docx |
| Q29 | Gemini | April 1-21, 2026 (query window) | Not prospectively archived | Gemini web (official; no API) | No documented resubmission | Raw_Gemini.docx |
| Q29 | Copilot | April 1-21, 2026 (query window) | Not prospectively archived | Microsoft Copilot web (official; no API) | No documented resubmission | Raw_Copilot.docx |
| Q29 | DeepSeek | April 1-21, 2026 (query window) | Not prospectively archived | DeepSeek web (official; no API) | No documented resubmission | Raw_DeepSeek.docx |
| Q29 | Doubao | April 1-21, 2026 (query window) | Not prospectively archived | Doubao web (official; no API) | No documented resubmission | Raw_Doubao.docx |
| Q30 | ChatGPT | April 1-21, 2026 (query window) | Not prospectively archived | ChatGPT web (official; no API) | No documented resubmission | Raw_ChatGPT.docx |
| Q30 | Gemini | April 1-21, 2026 (query window) | Not prospectively archived | Gemini web (official; no API) | No documented resubmission | Raw_Gemini.docx |
| Q30 | Copilot | April 1-21, 2026 (query window) | Not prospectively archived | Microsoft Copilot web (official; no API) | No documented resubmission | Raw_Copilot.docx |
| Q30 | DeepSeek | April 1-21, 2026 (query window) | Not prospectively archived | DeepSeek web (official; no API) | No documented resubmission | Raw_DeepSeek.docx |
| Q30 | Doubao | April 1-21, 2026 (query window) | Not prospectively archived | Doubao web (official; no API) | No documented resubmission | Raw_Doubao.docx |
| Q31 | ChatGPT | April 1-21, 2026 (query window) | Not prospectively archived | ChatGPT web (official; no API) | No documented resubmission | Raw_ChatGPT.docx |
| Q31 | Gemini | April 1-21, 2026 (query window) | Not prospectively archived | Gemini web (official; no API) | No documented resubmission | Raw_Gemini.docx |
| Q31 | Copilot | April 1-21, 2026 (query window) | Not prospectively archived | Microsoft Copilot web (official; no API) | No documented resubmission | Raw_Copilot.docx |
| Q31 | DeepSeek | April 1-21, 2026 (query window) | Not prospectively archived | DeepSeek web (official; no API) | No documented resubmission | Raw_DeepSeek.docx |
| Q31 | Doubao | April 1-21, 2026 (query window) | Not prospectively archived | Doubao web (official; no API) | No documented resubmission | Raw_Doubao.docx |
| Q32 | ChatGPT | April 1-21, 2026 (query window) | Not prospectively archived | ChatGPT web (official; no API) | No documented resubmission | Raw_ChatGPT.docx |
| Q32 | Gemini | April 1-21, 2026 (query window) | Not prospectively archived | Gemini web (official; no API) | No documented resubmission | Raw_Gemini.docx |
| Q32 | Copilot | April 1-21, 2026 (query window) | Not prospectively archived | Microsoft Copilot web (official; no API) | No documented resubmission | Raw_Copilot.docx |
| Q32 | DeepSeek | April 1-21, 2026 (query window) | Not prospectively archived | DeepSeek web (official; no API) | No documented resubmission | Raw_DeepSeek.docx |
| Q32 | Doubao | April 1-21, 2026 (query window) | Not prospectively archived | Doubao web (official; no API) | No documented resubmission | Raw_Doubao.docx |
| Q33 | ChatGPT | April 1-21, 2026 (query window) | Not prospectively archived | ChatGPT web (official; no API) | No documented resubmission | Raw_ChatGPT.docx |
| Q33 | Gemini | April 1-21, 2026 (query window) | Not prospectively archived | Gemini web (official; no API) | No documented resubmission | Raw_Gemini.docx |
| Q33 | Copilot | April 1-21, 2026 (query window) | Not prospectively archived | Microsoft Copilot web (official; no API) | No documented resubmission | Raw_Copilot.docx |
| Q33 | DeepSeek | April 1-21, 2026 (query window) | Not prospectively archived | DeepSeek web (official; no API) | No documented resubmission | Raw_DeepSeek.docx |
| Q33 | Doubao | April 1-21, 2026 (query window) | Not prospectively archived | Doubao web (official; no API) | No documented resubmission | Raw_Doubao.docx |
| Q34 | ChatGPT | April 1-21, 2026 (query window) | Not prospectively archived | ChatGPT web (official; no API) | No documented resubmission | Raw_ChatGPT.docx |
| Q34 | Gemini | April 1-21, 2026 (query window) | Not prospectively archived | Gemini web (official; no API) | No documented resubmission | Raw_Gemini.docx |
| Q34 | Copilot | April 1-21, 2026 (query window) | Not prospectively archived | Microsoft Copilot web (official; no API) | No documented resubmission | Raw_Copilot.docx |
| Q34 | DeepSeek | April 1-21, 2026 (query window) | Not prospectively archived | DeepSeek web (official; no API) | No documented resubmission | Raw_DeepSeek.docx |
| Q34 | Doubao | April 1-21, 2026 (query window) | Not prospectively archived | Doubao web (official; no API) | No documented resubmission | Raw_Doubao.docx |
| Q35 | ChatGPT | April 1-21, 2026 (query window) | Not prospectively archived | ChatGPT web (official; no API) | No documented resubmission | Raw_ChatGPT.docx |
| Q35 | Gemini | April 1-21, 2026 (query window) | Not prospectively archived | Gemini web (official; no API) | No documented resubmission | Raw_Gemini.docx |
| Q35 | Copilot | April 1-21, 2026 (query window) | Not prospectively archived | Microsoft Copilot web (official; no API) | No documented resubmission | Raw_Copilot.docx |
| Q35 | DeepSeek | April 1-21, 2026 (query window) | Not prospectively archived | DeepSeek web (official; no API) | No documented resubmission | Raw_DeepSeek.docx |
| Q35 | Doubao | April 1-21, 2026 (query window) | Not prospectively archived | Doubao web (official; no API) | No documented resubmission | Raw_Doubao.docx |
| Q36 | ChatGPT | April 1-21, 2026 (query window) | Not prospectively archived | ChatGPT web (official; no API) | No documented resubmission | Raw_ChatGPT.docx |
| Q36 | Gemini | April 1-21, 2026 (query window) | Not prospectively archived | Gemini web (official; no API) | No documented resubmission | Raw_Gemini.docx |
| Q36 | Copilot | April 1-21, 2026 (query window) | Not prospectively archived | Microsoft Copilot web (official; no API) | No documented resubmission | Raw_Copilot.docx |
| Q36 | DeepSeek | April 1-21, 2026 (query window) | Not prospectively archived | DeepSeek web (official; no API) | No documented resubmission | Raw_DeepSeek.docx |
| Q36 | Doubao | April 1-21, 2026 (query window) | Not prospectively archived | Doubao web (official; no API) | No documented resubmission | Raw_Doubao.docx |
| Q37 | ChatGPT | April 1-21, 2026 (query window) | Not prospectively archived | ChatGPT web (official; no API) | No documented resubmission | Raw_ChatGPT.docx |
| Q37 | Gemini | April 1-21, 2026 (query window) | Not prospectively archived | Gemini web (official; no API) | No documented resubmission | Raw_Gemini.docx |
| Q37 | Copilot | April 1-21, 2026 (query window) | Not prospectively archived | Microsoft Copilot web (official; no API) | No documented resubmission | Raw_Copilot.docx |
| Q37 | DeepSeek | April 1-21, 2026 (query window) | Not prospectively archived | DeepSeek web (official; no API) | No documented resubmission | Raw_DeepSeek.docx |
| Q37 | Doubao | April 1-21, 2026 (query window) | Not prospectively archived | Doubao web (official; no API) | No documented resubmission | Raw_Doubao.docx |
| Q38 | ChatGPT | April 1-21, 2026 (query window) | Not prospectively archived | ChatGPT web (official; no API) | No documented resubmission | Raw_ChatGPT.docx |
| Q38 | Gemini | April 1-21, 2026 (query window) | Not prospectively archived | Gemini web (official; no API) | No documented resubmission | Raw_Gemini.docx |
| Q38 | Copilot | April 1-21, 2026 (query window) | Not prospectively archived | Microsoft Copilot web (official; no API) | No documented resubmission | Raw_Copilot.docx |
| Q38 | DeepSeek | April 1-21, 2026 (query window) | Not prospectively archived | DeepSeek web (official; no API) | No documented resubmission | Raw_DeepSeek.docx |
| Q38 | Doubao | April 1-21, 2026 (query window) | Not prospectively archived | Doubao web (official; no API) | No documented resubmission | Raw_Doubao.docx |
| Q39 | ChatGPT | April 1-21, 2026 (query window) | Not prospectively archived | ChatGPT web (official; no API) | No documented resubmission | Raw_ChatGPT.docx |
| Q39 | Gemini | April 1-21, 2026 (query window) | Not prospectively archived | Gemini web (official; no API) | No documented resubmission | Raw_Gemini.docx |
| Q39 | Copilot | April 1-21, 2026 (query window) | Not prospectively archived | Microsoft Copilot web (official; no API) | No documented resubmission | Raw_Copilot.docx |
| Q39 | DeepSeek | April 1-21, 2026 (query window) | Not prospectively archived | DeepSeek web (official; no API) | No documented resubmission | Raw_DeepSeek.docx |
| Q39 | Doubao | April 1-21, 2026 (query window) | Not prospectively archived | Doubao web (official; no API) | No documented resubmission | Raw_Doubao.docx |
| Q40 | ChatGPT | April 1-21, 2026 (query window) | Not prospectively archived | ChatGPT web (official; no API) | No documented resubmission | Raw_ChatGPT.docx |
| Q40 | Gemini | April 1-21, 2026 (query window) | Not prospectively archived | Gemini web (official; no API) | No documented resubmission | Raw_Gemini.docx |
| Q40 | Copilot | April 1-21, 2026 (query window) | Not prospectively archived | Microsoft Copilot web (official; no API) | No documented resubmission | Raw_Copilot.docx |
| Q40 | DeepSeek | April 1-21, 2026 (query window) | Not prospectively archived | DeepSeek web (official; no API) | No documented resubmission | Raw_DeepSeek.docx |
| Q40 | Doubao | April 1-21, 2026 (query window) | Not prospectively archived | Doubao web (official; no API) | No documented resubmission | Raw_Doubao.docx |
| Q41 | ChatGPT | April 1-21, 2026 (query window) | Not prospectively archived | ChatGPT web (official; no API) | No documented resubmission | Raw_ChatGPT.docx |
| Q41 | Gemini | April 1-21, 2026 (query window) | Not prospectively archived | Gemini web (official; no API) | No documented resubmission | Raw_Gemini.docx |
| Q41 | Copilot | April 1-21, 2026 (query window) | Not prospectively archived | Microsoft Copilot web (official; no API) | No documented resubmission | Raw_Copilot.docx |
| Q41 | DeepSeek | April 1-21, 2026 (query window) | Not prospectively archived | DeepSeek web (official; no API) | No documented resubmission | Raw_DeepSeek.docx |
| Q41 | Doubao | April 1-21, 2026 (query window) | Not prospectively archived | Doubao web (official; no API) | No documented resubmission | Raw_Doubao.docx |
| Q42 | ChatGPT | April 1-21, 2026 (query window) | Not prospectively archived | ChatGPT web (official; no API) | No documented resubmission | Raw_ChatGPT.docx |
| Q42 | Gemini | April 1-21, 2026 (query window) | Not prospectively archived | Gemini web (official; no API) | No documented resubmission | Raw_Gemini.docx |
| Q42 | Copilot | April 1-21, 2026 (query window) | Not prospectively archived | Microsoft Copilot web (official; no API) | No documented resubmission | Raw_Copilot.docx |
| Q42 | DeepSeek | April 1-21, 2026 (query window) | Not prospectively archived | DeepSeek web (official; no API) | No documented resubmission | Raw_DeepSeek.docx |
| Q42 | Doubao | April 1-21, 2026 (query window) | Not prospectively archived | Doubao web (official; no API) | No documented resubmission | Raw_Doubao.docx |
| Q43 | ChatGPT | April 1-21, 2026 (query window) | Not prospectively archived | ChatGPT web (official; no API) | No documented resubmission | Raw_ChatGPT.docx |
| Q43 | Gemini | April 1-21, 2026 (query window) | Not prospectively archived | Gemini web (official; no API) | No documented resubmission | Raw_Gemini.docx |
| Q43 | Copilot | April 1-21, 2026 (query window) | Not prospectively archived | Microsoft Copilot web (official; no API) | No documented resubmission | Raw_Copilot.docx |
| Q43 | DeepSeek | April 1-21, 2026 (query window) | Not prospectively archived | DeepSeek web (official; no API) | No documented resubmission | Raw_DeepSeek.docx |
| Q43 | Doubao | April 1-21, 2026 (query window) | Not prospectively archived | Doubao web (official; no API) | No documented resubmission | Raw_Doubao.docx |
| Q44 | ChatGPT | April 1-21, 2026 (query window) | Not prospectively archived | ChatGPT web (official; no API) | No documented resubmission | Raw_ChatGPT.docx |
| Q44 | Gemini | April 1-21, 2026 (query window) | Not prospectively archived | Gemini web (official; no API) | No documented resubmission | Raw_Gemini.docx |
| Q44 | Copilot | April 1-21, 2026 (query window) | Not prospectively archived | Microsoft Copilot web (official; no API) | No documented resubmission | Raw_Copilot.docx |
| Q44 | DeepSeek | April 1-21, 2026 (query window) | Not prospectively archived | DeepSeek web (official; no API) | No documented resubmission | Raw_DeepSeek.docx |
| Q44 | Doubao | April 1-21, 2026 (query window) | Not prospectively archived | Doubao web (official; no API) | No documented resubmission | Raw_Doubao.docx |
| Q45 | ChatGPT | April 1-21, 2026 (query window) | Not prospectively archived | ChatGPT web (official; no API) | No documented resubmission | Raw_ChatGPT.docx |
| Q45 | Gemini | April 1-21, 2026 (query window) | Not prospectively archived | Gemini web (official; no API) | No documented resubmission | Raw_Gemini.docx |
| Q45 | Copilot | April 1-21, 2026 (query window) | Not prospectively archived | Microsoft Copilot web (official; no API) | No documented resubmission | Raw_Copilot.docx |
| Q45 | DeepSeek | April 1-21, 2026 (query window) | Not prospectively archived | DeepSeek web (official; no API) | No documented resubmission | Raw_DeepSeek.docx |
| Q45 | Doubao | April 1-21, 2026 (query window) | Not prospectively archived | Doubao web (official; no API) | No documented resubmission | Raw_Doubao.docx |
| Q46 | ChatGPT | April 1-21, 2026 (query window) | Not prospectively archived | ChatGPT web (official; no API) | No documented resubmission | Raw_ChatGPT.docx |
| Q46 | Gemini | April 1-21, 2026 (query window) | Not prospectively archived | Gemini web (official; no API) | No documented resubmission | Raw_Gemini.docx |
| Q46 | Copilot | April 1-21, 2026 (query window) | Not prospectively archived | Microsoft Copilot web (official; no API) | No documented resubmission | Raw_Copilot.docx |
| Q46 | DeepSeek | April 1-21, 2026 (query window) | Not prospectively archived | DeepSeek web (official; no API) | No documented resubmission | Raw_DeepSeek.docx |
| Q46 | Doubao | April 1-21, 2026 (query window) | Not prospectively archived | Doubao web (official; no API) | No documented resubmission | Raw_Doubao.docx |
| Q47 | ChatGPT | April 1-21, 2026 (query window) | Not prospectively archived | ChatGPT web (official; no API) | No documented resubmission | Raw_ChatGPT.docx |
| Q47 | Gemini | April 1-21, 2026 (query window) | Not prospectively archived | Gemini web (official; no API) | No documented resubmission | Raw_Gemini.docx |
| Q47 | Copilot | April 1-21, 2026 (query window) | Not prospectively archived | Microsoft Copilot web (official; no API) | No documented resubmission | Raw_Copilot.docx |
| Q47 | DeepSeek | April 1-21, 2026 (query window) | Not prospectively archived | DeepSeek web (official; no API) | No documented resubmission | Raw_DeepSeek.docx |
| Q47 | Doubao | April 1-21, 2026 (query window) | Not prospectively archived | Doubao web (official; no API) | No documented resubmission | Raw_Doubao.docx |
| Q48 | ChatGPT | April 1-21, 2026 (query window) | Not prospectively archived | ChatGPT web (official; no API) | No documented resubmission | Raw_ChatGPT.docx |
| Q48 | Gemini | April 1-21, 2026 (query window) | Not prospectively archived | Gemini web (official; no API) | No documented resubmission | Raw_Gemini.docx |
| Q48 | Copilot | April 1-21, 2026 (query window) | Not prospectively archived | Microsoft Copilot web (official; no API) | No documented resubmission | Raw_Copilot.docx |
| Q48 | DeepSeek | April 1-21, 2026 (query window) | Not prospectively archived | DeepSeek web (official; no API) | No documented resubmission | Raw_DeepSeek.docx |
| Q48 | Doubao | April 1-21, 2026 (query window) | Not prospectively archived | Doubao web (official; no API) | No documented resubmission | Raw_Doubao.docx |
| Q49 | ChatGPT | April 1-21, 2026 (query window) | Not prospectively archived | ChatGPT web (official; no API) | No documented resubmission | Raw_ChatGPT.docx |
| Q49 | Gemini | April 1-21, 2026 (query window) | Not prospectively archived | Gemini web (official; no API) | No documented resubmission | Raw_Gemini.docx |
| Q49 | Copilot | April 1-21, 2026 (query window) | Not prospectively archived | Microsoft Copilot web (official; no API) | No documented resubmission | Raw_Copilot.docx |
| Q49 | DeepSeek | April 1-21, 2026 (query window) | Not prospectively archived | DeepSeek web (official; no API) | No documented resubmission | Raw_DeepSeek.docx |
| Q49 | Doubao | April 1-21, 2026 (query window) | Not prospectively archived | Doubao web (official; no API) | No documented resubmission | Raw_Doubao.docx |
| Q50 | ChatGPT | April 1-21, 2026 (query window) | Not prospectively archived | ChatGPT web (official; no API) | No documented resubmission | Raw_ChatGPT.docx |
| Q50 | Gemini | April 1-21, 2026 (query window) | Not prospectively archived | Gemini web (official; no API) | No documented resubmission | Raw_Gemini.docx |
| Q50 | Copilot | April 1-21, 2026 (query window) | Not prospectively archived | Microsoft Copilot web (official; no API) | No documented resubmission | Raw_Copilot.docx |
| Q50 | DeepSeek | April 1-21, 2026 (query window) | Not prospectively archived | DeepSeek web (official; no API) | No documented resubmission | Raw_DeepSeek.docx |
| Q50 | Doubao | April 1-21, 2026 (query window) | Not prospectively archived | Doubao web (official; no API) | No documented resubmission | Raw_Doubao.docx |
| Q51 | ChatGPT | April 1-21, 2026 (query window) | Not prospectively archived | ChatGPT web (official; no API) | No documented resubmission | Raw_ChatGPT.docx |
| Q51 | Gemini | April 1-21, 2026 (query window) | Not prospectively archived | Gemini web (official; no API) | No documented resubmission | Raw_Gemini.docx |
| Q51 | Copilot | April 1-21, 2026 (query window) | Not prospectively archived | Microsoft Copilot web (official; no API) | No documented resubmission | Raw_Copilot.docx |
| Q51 | DeepSeek | April 1-21, 2026 (query window) | Not prospectively archived | DeepSeek web (official; no API) | No documented resubmission | Raw_DeepSeek.docx |
| Q51 | Doubao | April 1-21, 2026 (query window) | Not prospectively archived | Doubao web (official; no API) | No documented resubmission | Raw_Doubao.docx |
| Q52 | ChatGPT | April 1-21, 2026 (query window) | Not prospectively archived | ChatGPT web (official; no API) | No documented resubmission | Raw_ChatGPT.docx |
| Q52 | Gemini | April 1-21, 2026 (query window) | Not prospectively archived | Gemini web (official; no API) | No documented resubmission | Raw_Gemini.docx |
| Q52 | Copilot | April 1-21, 2026 (query window) | Not prospectively archived | Microsoft Copilot web (official; no API) | No documented resubmission | Raw_Copilot.docx |
| Q52 | DeepSeek | April 1-21, 2026 (query window) | Not prospectively archived | DeepSeek web (official; no API) | No documented resubmission | Raw_DeepSeek.docx |
| Q52 | Doubao | April 1-21, 2026 (query window) | Not prospectively archived | Doubao web (official; no API) | No documented resubmission | Raw_Doubao.docx |
| Q53 | ChatGPT | April 1-21, 2026 (query window) | Not prospectively archived | ChatGPT web (official; no API) | No documented resubmission | Raw_ChatGPT.docx |
| Q53 | Gemini | April 1-21, 2026 (query window) | Not prospectively archived | Gemini web (official; no API) | No documented resubmission | Raw_Gemini.docx |
| Q53 | Copilot | April 1-21, 2026 (query window) | Not prospectively archived | Microsoft Copilot web (official; no API) | No documented resubmission | Raw_Copilot.docx |
| Q53 | DeepSeek | April 1-21, 2026 (query window) | Not prospectively archived | DeepSeek web (official; no API) | No documented resubmission | Raw_DeepSeek.docx |
| Q53 | Doubao | April 1-21, 2026 (query window) | Not prospectively archived | Doubao web (official; no API) | No documented resubmission | Raw_Doubao.docx |

Abbreviations: API, application programming interface; Q, question. Exact model-question-level timestamps were not prospectively archived and are therefore reported as unavailable rather than retrospectively reconstructed. This query log should be interpreted together with Supplementary Material 5, which provides the cleaned full-text response record for all 265 model outputs.
