# Supplementary material for: Safety and quality of public chatbots for lung cancer prognostic information: a comparative evaluation
Source: Front Public Health. 2026 Aug 24;14:1935121. doi: 10.3389/fpubh.2026.1935121 (PMC13547269; doi:10.3389/fpubh.2026.1935121)
Supplement: Supplementary file 7 [file Table__7.docx]

Supplementary Material 7. Professional Characteristics and Expertise of the Independent Raters

**Description: This file summarizes the professional characteristics, clinical expertise, and relevant evaluation roles of the independent raters who assessed chatbot responses.**

| **Rater ID** | **Professional background** | **Academic qualification / academic role** | **Professional title and years of experience** | **Main expertise relevant to this study** |
| --- | --- | --- | --- | --- |
| Rater 1 | Thoracic surgery lung cancer oncology expert | Doctoral degree; master's supervisor | Chief physician; 26 years of clinical experience | Oncology research and lung cancer diagnosis, treatment, and prognostic evaluation |
| Rater 2 | Senior thoracic surgery lung cancer oncology expert | Master's supervisor | Chief physician; 34 years of clinical experience | Oncology research and senior expertise in lung cancer treatment and prognostic communication |
| Rater 3 | Health communication expert with oncology-related experience | Master's degree; master's supervisor | Chief Nurse; 26 years of professional experience | Oncology research and health communication; patient-facing risk communication and education |
| Rater 4 | Thoracic surgery lung cancer oncology expert | Postdoctoral qualification | Chief physician; 15 years of clinical experience | Oncology research and evidence-based medicine; lung cancer treatment and evidence appraisal |
| Rater 5 | Thoracic surgery lung cancer oncology nursing expert | Bachelor’s degree in nursing | Associate Chief Nurse; 28 years of professional experience | Lung cancer oncology nursing, perioperative care, patient education, and patient self-care management |

Note:In this table, Chief Nurse and Associate Chief Nurse refer to senior professional nursing titles rather than administrative positions.
