# Supplementary material for: Safety and quality of public chatbots for lung cancer prognostic information: a comparative evaluation
Source: Front Public Health. 2026 Aug 24;14:1935121. doi: 10.3389/fpubh.2026.1935121 (PMC13547269; doi:10.3389/fpubh.2026.1935121)
Supplement: Supplementary file 8 [file Table__8.docx]

Supplementary Material 8. Pairwise Comparisons of Safety

**Description: This file provides the pairwise safety comparisons across the five large language model chatbots, including adjusted statistical results and related comparison information.**

| **Comparison** | ***N* pairs** | **Safe rate difference, %** | **Safe rate difference 95% *CI*** | **Safe to unsafe, *n*** | **Unsafe to safe, *n*** | **Matched *OR*** | **Matched *OR* 95% *CI*** | **Adjusted *P*** | **Note** |
| --- | --- | --- | --- | --- | --- | --- | --- | --- | --- |
| ChatGPT vs Gemini | 53 | -11.32 | -24.53-1.89 | 4 | 10 | 0.40 | 0.13-1.28 | 0.302 | Pairwise McNemar test |
| ChatGPT vs Copilot | 53 | 1.89 | -11.32-15.09 | 8 | 7 | 1.14 | 0.41-3.15 | 1.000 | Pairwise McNemar test |
| ChatGPT vs DeepSeek | 53 | 16.98 | 1.89-32.08 | 14 | 5 | 2.80 | 1.01-7.77 | 0.200 | Pairwise McNemar test |
| ChatGPT vs Doubao | 53 | 9.43 | -7.59-26.42 | 14 | 9 | 1.56 | 0.67-3.59 | 0.558 | Pairwise McNemar test |
| Gemini vs Copilot | 53 | 13.21 | 0.00-28.30 | 11 | 4 | 2.75 | 0.88-8.64 | 0.243 | Pairwise McNemar test |
| Gemini vs DeepSeek | 53 | 28.30 | 13.21-43.40 | 18 | 3 | 6.00 | 1.77-20.37 | 0.023 | Pairwise McNemar test |
| Gemini vs Doubao | 53 | 20.75 | 5.66-35.85 | 15 | 4 | 3.75 | 1.24-11.30 | 0.109 | Pairwise McNemar test |
| Copilot vs DeepSeek | 53 | 15.09 | 0.00-28.30 | 12 | 4 | 3.00 | 0.97-9.30 | 0.200 | Pairwise McNemar test |
| Copilot vs Doubao | 53 | 7.55 | -9.43-24.53 | 12 | 8 | 1.50 | 0.61-3.67 | 0.558 | Pairwise McNemar test |
| DeepSeek vs Doubao | 53 | -7.55 | -22.64-7.55 | 7 | 11 | 0.64 | 0.25-1.64 | 0.558 | Pairwise McNemar test |

Note: Pairwise comparisons were performed using McNemar tests with Benjamini-Hochberg correction. Safe-rate differences and their bootstrap 95% *CIs* are reported.
